# The construction of modular universal chimeric antigen receptor T (MU-CAR-T) cells by covalent linkage of allogeneic T cells and various antibody fragments

**DOI:** 10.1186/s12943-024-01938-8

**Published:** 2024-03-11

**Authors:** Tao Chen, Jieyi Deng, Yongli Zhang, Bingfeng Liu, Ruxin Liu, Yiqiang Zhu, Mo Zhou, Yingtong Lin, Baijin Xia, Keming Lin, Xiancai Ma, Hui Zhang

**Affiliations:** 1 Institute of Human Virology, Department of Pathogen Biology and Biosecurity, Key Laboratory of Tropical Disease Control of Ministry Education, Guangdong Engineering Research Center for Antimicrobial Agent and Immunotechnology, Zhongshan School of Medicine, Sun Yat-sen University, Guangzhou, 510080 China; 2 Guangzhou National Laboratory, Guangzhou International Bio-Island, Guangzhou, 510005 China; 3 State Key Laboratory of Respiratory Disease, National Clinical Research Center for Respiratory Disease, Guangzhou Institute of Respiratory Health, the First Affiliated Hospital of Guangzhou Medical University, Guangzhou, 511400 China

**Keywords:** Universal CAR-T, Modular, SDcatcher/GVoptiTag, HIV-1, T cell lymphoma

## Abstract

**Background:**

Chimeric antigen receptor-T (CAR-T) cells therapy is one of the novel immunotherapeutic approaches with significant clinical success. However, their applications are limited because of long preparation time, high cost, and interpersonal variations. Although the manufacture of universal CAR-T (U-CAR-T) cells have significantly improved, they are still not a stable and unified cell bank.

**Methods:**

Here, we tried to further improve the convenience and flexibility of U-CAR-T cells by constructing novel modular universal CAR-T (MU-CAR-T) cells. For this purpose, we initially screened healthy donors and cultured their T cells to obtain a higher proportion of stem cell-like memory T (T_SCM_) cells, which exhibit robust self-renewal capacity, sustainability and cytotoxicity. To reduce the alloreactivity, the T cells were further edited by double knockout of the T cell receptor (TCR) and class I human leukocyte antigen (HLA-I) genes utilizing the CRISPR/Cas9 system. The well-growing and genetically stable universal cells carrying the CAR-moiety were then stored as a stable and unified cell bank. Subsequently, the SDcatcher/GVoptiTag system, which generate an isopeptide bond, was used to covalently connect the purified scFvs of antibody targeting different antigens to the recovered CAR-T cells.

**Results:**

The resulting CAR-T cells can perform different functions by specifically targeting various cells, such as the eradication of human immunodeficiency virus type 1 (HIV-1)-latenly-infected cells or elimination of T lymphoma cells, with similar efficiency as the traditional CAR-T cells did.

**Conclusion:**

Taken together, our strategy allows the production of CAR-T cells more modularization, and makes the quality control and pharmaceutic manufacture of CAR-T cells more feasible.

**Supplementary Information:**

The online version contains supplementary material available at 10.1186/s12943-024-01938-8.

## Background

The CAR-T cell therapy represents a cutting-edge and revolutionary immunotherapy approach. It has demonstrated remarkable clinical efficacy, particularly in treating hematological malignancies, thus being treated as one of the most promising anti-tumor therapies [1–4]. This approach also has been proposed as a therapeutic strategy to treat viral infections including HIV-1, hepatitis B virus (HBV), hepatitis C virus (HCV) and SARS-CoV-2 [5–8]. However, the production of conventional CAR-T cells is individually customized and not conducive to large-scale manufacturing, resulting in prolonged preparation time for patients [9]. Consequently, many patients with advanced or rapidly progressing diseases often present at a stage when treatment options are limited [10]. Meanwhile, the efficacy and durability of CAR-T cell therapy within patients are hampered by various challenges including tumor antigen heterogeneity, immunosuppressive tumor microenvironment, antigenic escape and T cells exhaustion [11]. Therefore, universal CAR-T cells therapies that can simultaneously target multiple antigens or efficiently target pan-epitopes are promising alternative approaches [12].

The development of modular CAR-T cells systems is accelerating, enabling precise control of CAR-T cells through adapters to separate the T cell signaling domain and the antigen-recognition domain, unlike traditional intact CAR-T cells [13]. The adapter systems are also the focuses of universal CAR-T cells research currently, such as BBIR CAR [14], anti-GCN4 CAR, anti-FITC CAR [15], Spy CAR system [16], Uni CAR [17], SUPRA CAR [18], Convertible CAR [19, 20] and BsAb adapter CAR [21]. These various adapters-conjugated approaches, most of them are not covalent connections, allow T cells redirection by swapping different single-chain variable fragments (scFvs) without frequent CAR replacement and time-consuming remanufacturing. As a result of their flexibility, these methods significantly enhance their clinical applicability (NCT04450069, NCT02776813 and NCT04633148).

The maintenance of long-term survival and proliferation capacity of T cells in vivo are crucial for achieving sustained efficacy of CAR-T therapy. T_SCM_ cells are rare lymphocyte subsets with long-term survival ability and strong potential for immune reconstitution [22]. In preclinical animal models, adoptively transferred T_SCM_-like CAR-T cells exhibit potent proliferation and anti-tumor abilities [23, 24]. In addition, patients who response better to CD19-directed CAR-T therapy also have an increased frequency of memory CAR-T cells within their CAR-T reservoirs [25]. Therefore, the proportion of T_SCM_ subsets in the expanded T cells is correlated to the therapeutic effect of CAR-T therapy.

In this study, we successfully constructed a MU-CAR-T model based on the SDCatcher/GVoptiTag (Sd/Gv) covalent attachment system developed in our laboratory previously [26]. ScFvs targeting diversified antigens were covalently conjugated to T cells carrying CAR moiety, resulting in the efficient suppression of infection and tumor progression. To enhance the efficacy of universal CAR-T cells, we screened and optimized T cells to obtain highly plastic T cells with superior expansion ability and more robust functionality. Furthermore, to enhance the safety of CAR-T cells, the allogeneically T cells were gene-edited by CRISPR/Cas9 technology to eliminate the expression of the endogenous TCR, thus avoiding the graft-versus-host disease (GVHD). Besides, the expression of the HLA-I was also aborted by CRISPR/Cas9-mediated knockout to avoid host-versus-graft reaction. TCR and HLA-I double-negative T cells were sorted to prepare stable universal CAR-T cell storages. Finally, the constructed MU-CAR-T cells were successfully validated for their therapeutic effects in anti-infection and anti-tumor utilizing both in vitro and in vivo experiments, which was represented by VRC01 scFv-mediated efficient killing of latent HIV-1-infected cells and CD5-CD30 scFvs-mediated successful suppression of T cell lymphoma. Our results demonstrated that MU-CAR-T cells were able to target various antigens without the need to re-edit autologous or allogeneic T cells.

## Methods

### Cells lines and culture

HEK293T, HEK293T and Karpas 299 cells were obtained from ATCC. Jurkat_gp160_ cells were established by the infection of Jurkat cells with recombinant lentiviruses carrying HIV-1_NL4 − 3_ Env-IRES-GFP moiety, followed by sorting GFP^hi^ cells. HEK293T cells were cultured in Dulbecco’s modified Eagle medium (DMEM; Sigma-Aldrich, USA) supplemented with 10% fetal bovine serum (FBS, Gibco, USA). Jurkat_gp160_ and Karpas 299 were cultured in RPMI 1640 medium (Sigma-Aldrich, USA) plus 10% FBS. HEK293F cells were cultured in Unio-293 F (Union-Biotech, China) medium supplemented with 1% GlutaMAX (Gibco, USA) and 1% penicillin-streptomycin (Gibco, USA), and incubated in polycarbonate vent-cap Erlenmeyer shaker nask under 37 ℃, 8% CO_2_ and 110 rpm speed in an orbital shaker. All cells have been confirmed to be mycoplasma-free utilizing PCR-based methods.

### Construction of lentiviral vector

The 28BBZ3 structure served as an intracellular signaling domain for third-generation CAR-T cells, which consisted of the CD28 (nucleotides 460–660, GenBank NM_006139.3), 4-1BB (nucleotides 640–765, GenBank NM_001561.5), and CD3ζ (nucleotides 160–492, Genbank NM_198053.2) intracellular domains interconnected with a GGGGS sequence inserted between each domain. The signal peptide (SP) and SdΔN17 domain were fused to the 5’ end of 28BBZ3, while tCD19 was linked to the 3’ end of 28BBZ3 via P2A. This resulted in the construction of SP-SdΔN17-28BBZ3-P2A-tCD19, which was cloned into the pHR vector (Addgene#60,954) via MluI and NotI cloning sites. Meanwhile, we connected the VRC01 scFv and CD5-CD30 scFv sequences separately to the third-generation intracellular CAR moiety in order to construc conventional CAR-T cells as controls. All constructs were verified by sequencing, and the protein sequences of each construct are listed in Table S1.

### Protein expression and purification

Gv-GFP was expressed and purified from *Escherichia coli (E.coli)*. Briefly, the DNA sequences of *6*×*His-tagged Gv-GFP* were cloned to the pET28a vector. The construct was transformed into BL21 (TaKaRa, Japan). A single clone was amplified in lysogeny broth (LB) with 0.1% kanamycin in a shaking incubator (37 ℃, 220 rpm). Then Isopropyl b-D-1thiogalactopyranoside (IPTG, TaKaRa, Japan) was then added to the bacteria solution with a final concentration of 1 mM to induce the protein expression (16 ℃, 220 rpm). After 18 h of induction, the bacteria were collected and lysed by sonication. The cleared supernatant was incubated with Ni-NTA agarose (Cytiva, USA) to enrich His-labeled Gv-GFP proteins. Proteins were subsequently eluted utilizing Tris buffer containing imidazole. Purified proteins were concentrated and exchanged with conventional Tris buffer to remove residue imidazole. The BCA method was used to determine the concentration of Gv-GFP proteins.

Gv-VRC01 scFv and Gv-CD5-CD30 scFvs were expressed and purified from HEK293F. Briefly, the N-termini of *scFvs* were fused by the sequences of secretory signal peptide, while the C-termini were followed by the sequences of *Gv-6×His*. DNA sequences were cloned into pcDNA3.1 vector. These constructs were transfected into HEK293F cells. After 7 days, the supernatants were collected and centrifuged to remove cellular debris. The purified supernatant was passed through Ni-NTA agarose to enrich His-tagged target proteins and then eluted with Tris buffer containing imidazole. Eluted proteins were concentrated and buffer-replaced with conventional Tris buffer. The concentration was determined utilizing the BCA assay. Finally, the purity of these proteins was confirmed through Coomassie blue staining and western blotting against His tag.

### Isolation and culture of human primary T lymphocytes

Human peripheral blood mononuclear cells (PBMCs) derived from healthy donors were isolated from buffy coats by Ficoll-Hypaque gradient separation. For the selection of optimized medium, sorted PBMCs were resuspended in different media (RPMI 1640, Sigma-Aldrich, USA; KBM581, Conring, USA; X-VIVO15, Lonza, Switzerland; OptiVitro T-SFM, ExCell, China; and ImmunoCult-XF, STEMCELL, Canada) at a density of 10^6^ cells /mL. These cells were stimulated for 2 days with anti-CD3 antibodies at 1 µg/mL (STEMCELL, Canada), anti-CD28 antibodies (STEMCELL, Canada) at 1 µg/mL, and recombinant human interleukin-2 (IL-2) at 10 ng/mL (Proteintech, USA). Then, the cell morphology was characterized and the number was counted every 3 days, while the percentage of CD8^+^ T cells was detected utilizing anti-CD8 antibodies.

For the selection of cytokines, sorted PBMCs were resuspended in OptiVitro T-SFM serum-free medium at a density of 10^6^ cells/mL, and anti-CD3 and anti-CD28 antibodies with a final concentration of 1 µg/mL were added to activate cells. Cells were then cultured with IL-2 at 10 ng/mL, or IL-7 at 10 ng/mL (Proteintech, USA) and IL-15 at 5 ng/mL (Proteintech, USA). After 5 days of activation, cells were transferred to a shaker at a speed of 88 rpm/min for continuing culture. Cells were maintained at a density of 10^6^ cells/mL. The cell number and percentage of CD8^+^ T cells were measured every 3 days.

Human primary CD8^+^ T cells were obtained from the above PBMCs by negative magnetic selection utilizing human CD8^+^ T lymphocyte enrichment set DM (BD-IMag, USA). All cells have been tested for mycoplasma utilizing PCR assay and confirmed to be mycoplasma-free.

### Cryopreservation and thawing of T cells

CD8^+^ T cells were frozen in cryopreservation solution CryoStor CS10 (Biolife solutions, USA) at a concentration of 1 ~ 2 × 10^7^ cells/mL in 1 mL aliquots. Cells within freezing containers were allowed to freeze at -80 ℃ for 24 h prior to transferring into liquid nitrogen container. After one week of storage, frozen cells were rapidly thawed in a water bath at 37 ℃ and then washed in 10 mL of pre-warmed media. Cells were resuspended in medium to a concentration of 10^6^ cells/mL and detected the cell viability utilizing the Zombie Green Fixable Viability Kit.

### Gene editing validation

The disruption of *TRAC* and *B2M* genes in T cells was assessed by a T7 endonuclease I (T7E1) Surveyor Nuclease assay (NEB). Briefly, the genomic DNA from cells that had been knocked out of *TRAC* and *B2M* genes was collected and amplified using target-specific primers. PCR products were annealed, followed by the addition of 0.5 µL of T7E1 to the annealed products. The mixture was then digested at 37°C for 30 minutes and the digested bands were visualized utilizing 2% agarose gel electrophoresis. T7E1 can recognize and cleave the mismatched DNA which occurs within knockout DNA samples. In addition, some of PCR products were verified by Sanger sequencing, and the other part was ligated to the pMD18T vector (TaKaRa, Japan) and then transformed in *E.coli.* Single clone was picked and sequenced to confirm the indels and insertions. The PCR primers used for the amplification of the target locus were as follows: *TRAC* forward, 5’-TCATGTCCTAACCCTGATCCTCTT-3’; *TRAC* reverse, 5’-TTGGACTTTTCCCAGCTGACAGA-3’; *B2M* forward, 5’-TCATGTCCTAAAGCTGACAGCATTC-3’; *B2M* reverse, 5’-CTGTTTTCAAAATTAAATGACGC-3’.

### Generation and isolation of TCR^−^/HLA-I^−^ cells

*Cas9* mRNA and sgRNAs targeting *TRAC* and *B2M* were designed and synthesized (RiboBio, China). T cells were stimulated with anti-CD3 and anti-CD28 antibodies for three days before being electroporated according to the instructions of the P3 Primary Cell 4D-Nucleofector X kit (Lonza, Switzerland). Briefly, 5 × 10^6^ cells were resuspended in 100 µL of supplemented Nucleofector solution. The sgRNA and *Cas9* mRNA in a 1:1 ratio were added into the solution. The mixture was transferred to a Nucleocuvette and inserted into the 4D-Nucleofector X Unit instrument, followed by performing the electroporation program of E0-115. This process was followed by a second electroporation at 12 to 24 h later. Following electroporation, cells were immediately transferred to pre-warmed medium and cultured in the presence of IL-2 (10 ng/mL) at 37 °C and 5% CO_2_.

The population of TCR/HLA-I double-negative cells was sorted on Day 6. Cells were incubated with biotin-conjugated anti-TCR and biotin-conjugated anti-HLA-I antibodies (BioLegend, USA) for 30 min at 4 °C after being washed with PBS for three times. Cells were then incubated with streptavidin Nanobeads for 15 min. Target cells were collected from the supernatant through multiple attachments to the MojoSort magnet. The expression of TCR and HLA-I molecules was confirmed both before and after the sorting process.

### Enrichment of CD19-positive cells

CD8^+^ T cells overexpressing tCD19 (contain the extracellular and transmembrane domain of CD19) were washed with Auto MACS buffer and incubated with CD19 microbeads (Miltenyi Biotec, Germany) at 4 ℃ for 30 min. After being washed twice, cells were passed through an LS column (Miltenyi Biotec, Germany). The LS Column was then removed from the separator and transferred to a new collection tube. The fraction containing magnetically labeled cells was flushed out and collected for further use.

### LDH assay

The lactate dehydrogenase (LDH) assay was utilized to determine the specific killing activity of engineered T cells against Jurkat_gp160_ or Karpas 299 cells at different ratios (from 10:1 to 0.625:1) according to the manufacturer’s instructions (Promega, USA). Briefly, Target cells were co-cultured with effector cells in a 96-well U-bottom plate for 24 to 48 h. Wells containing only target cells were mixed with the lysis reagent and incubated for 30 min at 37 °C. After being mixed with the LDH reaction substrate for 30 min, the absorbance was measured at 490 nm. Absorbance values of wells containing effector cells alone and target cells alone were combined and subtracted as the background from the values of the co-cultures. The percentage of cytotoxicity was calculated utilizing the formula provided by the manufacturer.

### Enzyme-linked immunosorbent assay (ELISA)

Engineered T cells or control effector CD8^+^ T cells (10^5^ cells) were co-cultured with target cells at a 4:1 ratio in 96-well round-bottom plates. The supernatants were collected after 18 to 24 h. Cytokine release by effector CD8^+^ T cells was analyzed utilizing Granzyme B, IFN-γ and TNFα ELISA kits (Dakewe, China). Briefly, the supernatants were incubated within wells which were pre-coated with Granzyme B, IFN-γ and TNFα for 2 h at room temperature respectively. Then, the biotinylated primary antibodies were added and incubated at room temperature for 1 h. After washing for 3 times, streptavidin-HRP was added and incubated for 30 min at room temperature. After being mixed with the TMB reaction substrate for 5 minutes, the reaction was immediately stopped by adding stop solution and the absorbance within each well was measured at 450 nm. The production of HIV-1 viral particle in cell cultures was determined with an HIV-1 p24 ELISA kit following the manufacturer’s protocol (Clontech, USA).

### IFN-γ ELISPOT assay

The ELISPOT assays were performed by mixing engineered T cells or control effector CD8^+^ T cells (10^5^ cells) with target cells at a 4:1 effector-target ratio, and then added to the IFN-γ antibodies-precoated plates (Dakewe, China). Effector CD8^+^ T cells alone were treated as negative control, while phytohemagglutinin (PHA) stimulated CD8^+^ T cells were treated as positive control. Plates were incubated for 18 to 24 h at 37 °C and 5% CO_2_. For allogeneic reaction, TCR^−^/HLA-I^−^ T cells were co-cultured with irradiated allogenic PBMCs (60 Gy) at a concentration of 5 × 10^4^ cells per well in IFN-γ ELISPOT plates. In a parallel experiment, allogeneic PBMCs were co-cultured with TCR^−^/HLA^−^ T cells that had been irradiated. Two groups of cells were incubated in a 1:1 ratio for 18 h. The experiment was subsequently performed according to the manufacturer’s instructions. Briefly, cells were removed from the plates and plates were washed for 6 times with washing buffer. Then, biotinylated IFN-γ antibodies were added and incubated for 1 h at 37 °C. After washing for 6 times, streptavidin-HRP was added and incubated for 1 h at 37 °C. Subsequently, AEC buffer was added to each well and incubated for 10 min at room temperature in the dark. Plates were scanned and the number of positive spots was calculated using the ImmunoSpot S6 ultra analyzer (Cellular Technology Ltd., USA).

### In vitro wild-type HIV-1 infection and drug withdrawal model

CD4^+^ T cells which were derived from PBMCs of healthy donors were stimulated with 1 µg/mL of PHA and 10 ng/mL of IL-2 in a serum-free medium for 2 days. Activated cells were then infected with laboratory virus strain NL4-3 (p24 titer of 1 ng/mL). Three hours post-infection, the fresh medium containing 10 ng/mL of IL-2 was replaced via centrifugation. After six days of HIV-1_NL4 − 3_ infection, a combination of azidothymidine (AZT, Sigma-Aldrich, USA) and lopinavir (Sigma-Aldric, USA) at a concentration of 20 µM was administrated to inhibit viral replication and prevent further infection. Additionally, cells were cultured in low concentrations of IL-2 (1 ng/mL). Approximately 6 to 8 days post drug administration, the production of virus significantly decreased to the marginal level which was indicated by the p24 expression level. Subsequently, anti-HIV-1 drugs were withdrawn, and 0.5 × 10^6^ infected CD4^+^ T cells were co-cultured with various groups of CAR-T cells at a ratio of 1:4 in 24-well plates. The HIV-1 p24 antigen in the culture was monitored every 2 days utilizing the HIV-1 p24 antigen assay kit following the manufacturer’s instructions.

### Real-time qRT-PCR analysis

On Day 26 of in vitro wild-type HIV-1 infection and drug withdrawal experiments, HIV-infected CD4^+^ T cells were harvested. Total RNAs were extracted using TRIzol reagent (Life Technologies, USA) and then subjected to cDNA synthesis with PrimeScript reverse transcription (RT) reagent kit (TaKaRa, Japan). The SYBR PreMix Ex Taq II kit (TaKaRa, Japan) was used to perform quantitative PCR according to the manufacturer’s instructions. The quantification of viral RNAs was determined by real-time quantitative reverse transcription-PCR (qRT-PCR) with the primer pair SK38 (5’-ATAATCCACCTATCCCAGTAGGAGAAA-3’) and SK39 (5’-TTTGGTCCTTGTCTTATGTCCAGAATGC-3’). Expression quantification of target genes was normalized to the housekeeping gene *β-actin* (forward primer: 5’-GCATGGAGTCCTGTGGCA-3’; reverse primer: 5’-CAGGAGGAGCAATGATCTTGA-3’).

### Xenograft models

NCG mice (Strain NO. T001475) were purchased from GemPharmatech Co, Ltd (Nanjing, China) and housed in SPF facilities at the Laboratory Animal Center of Sun Yat-sen University. All mice (6–8 weeks old) were randomly divided into four groups, with five mice in each group. They were then injected with 3 × 10^6^ Karpas 299 lymphoma cells via subcutaneous injection on the right side of their backs. When the tumor volume reached approximately 100 mm^3^, various groups of CAR-T cells were administrated via the tail vein for therapeutic purposes. CAR-T cells were administrated at a dose of 6 × 10^6^ cells per mouse. The ethical end point of the experiment was defined as upon the diameter of the tumor exceeded 20 mm. Mice were euthanized before the ethical end point. Mice tumors were collected for follow-up experiments.

### Flow cytometry

The following fluorescent dye-conjugated antibodies or reagents were used to label cells: FITC-CD8 (Clone RPA-T8, Cat:301,050), PerCP-Cy5.5-CD25 (Clone BC96, Cat:302,625), PE-HLA-DR (Clone L243, Cat:307,605), Alexa Fluor 488-CCR7 (Clone G043H7, Cat:353,205), APC/Cy7-CD45RA (Clone HI100, Cat:304,127), APC-CD122 (Clone TU27, Cat:339,007), PE-CD95 (Clone DX2, Cat:305,607), PE-CD19 (Clone HIB19, Cat:302,208), APC-TCRα/β (Clone IP26, Cat:306,718), FITC-HLA-A, B, C (Clone W6/32, Cat:311,404) and Zombie Green Fixable Viability (all from BioLegend, USA).

For the detection of cytokines-expressing cells, mice tumors were firstly digested with collagenase (Sigma-Aldrich, USA). The infiltrating mononuclear cells were obtained using the tumor lymphocyte isolation kit (Solarbio, China). The Intracellular (IC) Fixation buffer (eBioscience, USA) was added into cell medium and incubated at room temperature for 2 h to fix cells. After centrifugation for 5 min, cells were resuspended in permeabilization buffer (eBioscience, USA) and incubated with PE-IFN-γ and Alexa Fluor 700-Granzyme-B antibodies (BioLegend, USA) at 4 ℃ for 20 min. All flow cytometry data were acquired on FACS Aria II flow cytometer (BD Biosciences, USA) and analyzed using FlowJo software.

### Immunofluorescence and immunohistochemistry

For immunofluorescence assay, HEK293T cells expressing Sd-28BBZ3-P2A tCD19 were incubated with His-tagged Gv-scFv proteins for 12 hours. Subsequently, cells were fixed with 4% paraformaldehyde for 10 minutes, permeabilized with 1% Triton X-100 for 10 minutes, and then incubated with 5% bovine serum albumin (BSA) for 10 minutes to block nonspecific binding. Cells were subsequently incubated with a mixture of PE-CD19 (BioLegend, USA) and FITC-His (BioLegend, USA) at a dilution of 1:200 for 2 hours at room temperature. 4’,6-Diamidino-2-phenylindole (DAPI) (Life Technologies, USA) was used for nuclear staining. Images were acquired using super-resolution Structured Illumination Microscopy (SIM) and analyzed with NIS-Elements AR software (Nikon, Japan).

 For immunohistochemistry assay, tumors were collected and fixed in 4% paraformaldehyde for 48 hours. Then the tissue was embedded in paraffin and sliced. Sections (3–4 µm) were deparaffinized and rehydrated with xylene and gradient alcohol. Antigen repair was performed using a microwave with citrate buffer (pH 6.0), followed by quenching of endogenous peroxidase for 10 minutes with 3% H_2_O_2_. The nonspecific binding site was blocked with BSA for 30 minutes at room temperature, and then samples were incubated overnight at 4 ℃ with rabbit anti-human CD8, IFN-γ and Granzyme B (Proteintech, USA) antibodies at a dilution of 1:200 respectively. Next, sections were incubated with HRP-conjugated goat anti-rabbit IgG for 2 hours at room temperature and stained with 3,3’-diaminobenzidine. Finally, sections were subjected to hematoxylin staining, followed by dehydration with gradient concentrations of ethanol and clearance with xylene. Image acquisition was performed using a microscope (BX63, Olympus, Japan).

### Statistical analysis

Statistical analyses were performed using GraphPad Prism software version 8.3. For comparison between two groups, the Student’s *t*-test was used. For multiple comparisons within a dataset containing more than two groups, one-way analysis of variance (ANOVA) with Tukey’s multiple comparisons test was used. For the CTL assay, cell expansion studies and the tumor growth experiment, two-way ANOVA was performed. Data were presented as mean ± SD. A value of *p* < 0.05 (represented as *) was considered to be statistically significant. Value of *p* < 0.01 (represented as **) was considered to be more statistically significant. Value of *p* < 0.001 (represented as ***) was considered to be the most statistically significant. Value of *p* < 0.0001 (represented as ****) was considered to be the extremely statistically significant.

## Results

### The design and functional validation of modular CAR based on the Sd/Gv system

**Fig. 1:**
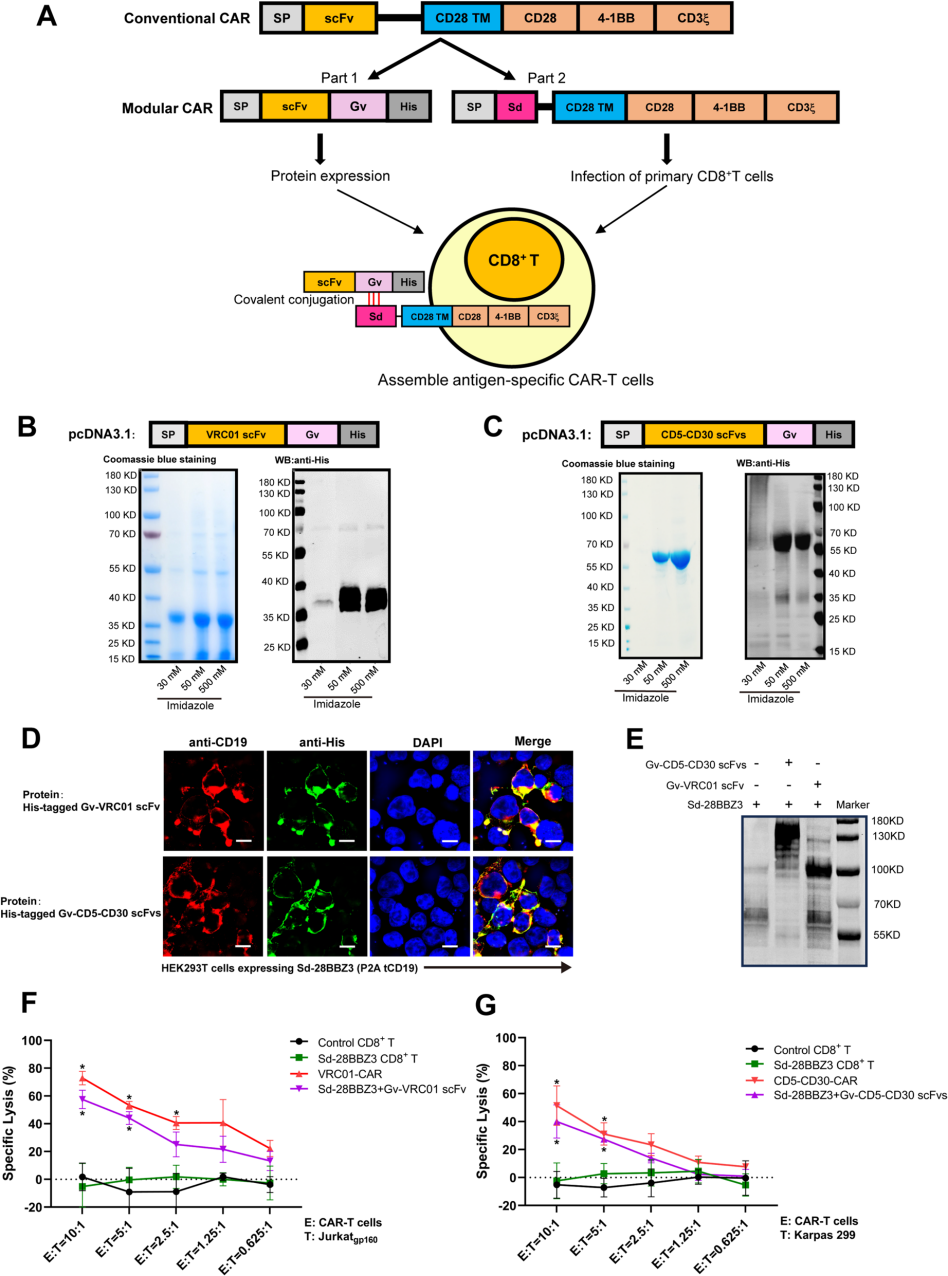
The Sd/Gv-based CAR effectively redirected T cells to eliminate target cells. **A** Schematic representation of the design of modular CAR by Sd/Gv platform. Sd: SDCatcher, Gv: GVoptiTag. **B** and **C** Coomassie blue staining and western blotting analysis of Gv-VRC01 scFv and Gv-CD5-CD30 scFvs. Anti-His antibody was used to confirm the expression and purity of each protein. **D** The Sd-28BBZ3-P2A-tCD19 elements were transduced into HEK293T cells, and the expression of CD19 indirectly indicated the expression of Sd-28BBZ3. Meanwhile, Gv-scFvs (VRC01 scFv or CD5-CD30 scFvs) were expressed as fusion proteins with a 6-Histidine tag. The cells expressing Sd-28BBZ3-P2A-tCD19 were incubated with Gv-scFv proteins, allowing for indirect detection of Gv-scFV binding to Sd on the cell surface through the presence of His tag. Immunofluorescence staining analysis of His-tagged Gv-scFv proteins conjugating to Sd-28BBZ3-P2A-tCD19 on HEK293T cells with anti-CD19 (red) and anti-His (green) antibodies. DAPI was used to stain the nuclei (blue). Scale bars represented 10 μm. **E** The Sd-28BBZ3-P2A-tCD19 elements were transduced into HEK293T cells. After 12 h, the culture medium was removed and the cells were cultured with fresh medium supplemented with 100 nM Gv-scFVs (VRC01 scFv or CD5-CD30 scFvs) proteins. Following an additional 12 h, the membrane proteins were extracted and the covalent binding of Sd-Gv was detected using western blot with anti-Gv/Sd antibodies. **F** and **G** Direct killing of target cell lines was performed utilizing the Cyto-Tox nonradioactive cytotoxicity kit with Jurkat_gp160_ (**F**) or Karpas 299 (**G**) cells as targets. The effector cells included the following cells. Control CD8^+^ T: CD8^+^ T cells transduced with empty vector. Sd-28BBZ3 CD8^+^ T: CD8^+^ T cells transduced with Sd-28BBZ3. Sd-28BBZ3 + Gv-scFv: CD8^+^ T cells transduced with Sd-28BBZ3 and incubated with proteins of Gv-scFvs (VRC01 scFv or CD5-CD30 scFvs). VRC01-CAR: CD8^+^ T cells transduced with conventional VRC01-CAR. CD5-CD30-CAR: CD8^+^ T cells transduced with conventional CD5-CD30-CAR. Different ratios of effector cells and target cells were co-incubated. Data represented as mean ± SD. *N* = 3 independent biological replicates. Statistical analysis was performed by two-way ANOVA followed by Tukey’s multiple comparisons test. * *p* < 0.05, ** *p* < 0.01

Many Gram-positive bacteria in nature contain extracellular proteins stabilized by spontaneous in the efficacies of the MU-CAR-T cells have been demonstrated for tramolecular isopeptide bonds [27, 28]. We previously developed an Sd/Gv system derived from *Streptococcus dysgalactiae* and *Gardnerella vaginalis* respectively [26]. Sd/Gv system was characterized of more efficient covalent bond formation ability. In this study, we utilized this system to produce universal CAR-T cells (Fig. 1A, Supplementary Fig. 1A). The CAR moiety was divided into two parts which were the extracellular antigen-recognition domain and the intracellular signal transduction domain. The Gv-tagged scFv was characterized as an extracellular recognition element. The intracellular domain 28BBZ3 contained truncated CD28 and 4-1BB co-stimulatory domains, along with an immunoreceptor tyrosine-based activation motifs (CD3ζ), as previously designed and published by our group [29]. The Sd domain, which could bind to the Gv tag, was connected to the intracellular domain 28BBZ3 (Supplementary Fig. 1B) [29]. The truncated CD19 (tCD19) protein was linked to 28BBZ3 by 2 A peptide (P2A), which served as the sorting marker. The resulting Sd-28BBZ3 was transduced into CD8^+^ T cells. Two extracellular antigen-recognition domains were chosen for multi-target validation. The VRC01 scFv was obtained from the broadly neutralizing HIV-1-specific antibody VRC01, and the bispecific CD5-CD30 scFvs were derived from the humanized CD5-specific antibody (H65) and the anti-CD30 antibody (brentuximabum). We fused these scFvs with Gv and purified them from HEK293F cells (Fig. 1B-C). Furthermore, to confirm that they do not interfere with each other’s functionality, we performed structural predictions of Gv-VRC01 scFv and Gv-CD5-CD30 scFvs using AlphaFold (Supplementary Fig. 2). The Gv-tagged scFvs were purified as proteins and characterized as an extracellular recognition element. The Sd-28BBZ3 was transduced into cells, followed by incubation with Gv-tagged scFvs proteins for 12 h. Through the Sd/Gv system, scFvs were able to be conjugated to the Sd-28BBZ3-expressing cells, resulting in the formation of complete MU-CAR via potent Sd-Gv covalent conjugation (Fig. 1D-E).

To determine the covalent loading and the binding stability, the binding efficiencies of Gv-tagged GFP (Gv-GFP) proteins at different concentrations were evaluated after co-incubating with Sd-28BBZ3-expressing CD8^+^ T cells for 12 h. The protein concentration with the highest binding efficiency was determined to be 100 nM (Supplementary Fig. 3A-B). We further monitored the binding stability for another 12 h after protein withdrawal. The results showed that the binding efficiency did not change significantly, which was represented by the stable maintenance of bound proteins on the cell surface (Supplementary Fig. 3C-D). Subsequently, we validated the function of modular CAR-T cells based on the Sd/Gv system. The HIV-1 precursor glycoprotein gp160 can be efficiently targeted by broadly neutralizing antibody (bnAb) VRC01, resulting in the neutralizing of various HIV-1 strains [30, 31]. CD5 is highly expressed in approximately 85% of T cell malignancies, while CD30 exhibits high expression in lymphomas, specifically in anaplastic large cell lymphoma (ALCL) [32, 33]. Both CD5 and CD30 are treated as classical targets for cancer therapy. Thus, HIV-1 gp160-expressing Jurkat (Jurkat_gp160_) cells and T lymphoma cell line Karpas 299 were selected as target cells for VRC01 scFv and CD5-CD30 scFvs respectively. Meanwhile, conventional VRC01 CAR and CD5-CD30 CAR were used as positive controls (Supplementary Fig. 3E). The cytotoxic potency in each group of T cells was evaluated by detecting the release of LDH. The results demonstrated that the Sd/Gv-based CAR effectively redirected T cells to eliminate target cells (Fig. 1F-G). Substituting different scFvs induced the death of specific target cells. VRC01 scFv-conjugated CAR-T cells effectively eliminated Jurkat_gp160_ cells, while CD5-CD30 scFvs-conjugated CAR-T cells efficiently killed Karpas 299 cells (Fig. 1F-G). Therefore, we have proved that the conventional CAR could be separated into two individual fragments, which could be coalesced into complete CAR again through the spontaneously covalent attachment of Sd and Gv. Importantly, the generated modular CAR-T cells exhibited potent cytotoxicity as conventional CAR-T cells in vitro.

### CRISPR/Cas9-mediated TCR and HLA-I disruption and reduction of allogeneic reactivity

The endogenous TCR presented on allogeneic T cells has the potential to recognize the alloantigen of the recipient, leading to the GVHD [34]. The human major histocompatibility complex (MHC) is a gene family that encodes human leukocyte antigens (HLAs). HLA molecules from donor cells are polymorphic antigens that can be recognized by recipient T cells, resulting in the histocompatibility rejection [35]. Thus, it is necessary to disrupt TCR and HLA-I on allogeneic T cells to eliminate the allogenic reaction of universal CAR-T cells. The TCRs are highly heterodimers composed of α chain (encoded by *TRAC* gene) and β chain (encoded by *TRBC* gene), which are expressed on most T cells [36]. Each polymorphic HLA class I protein forms a heterodimer with β 2-microglobulin (encoded by *B2M* gene), a genetically identical protein in humans, the heterodimer formation of which is required for HLA-I presentation on the cell surface [37, 38]. We chose to knock out the *TRAC* and *B2M* genes in order to disrupt TCR and HLA-I expression, respectively. This is a common method for generating universal CAR-T cells [39–41]. We initially screened potential sgRNAs in Jurkat cell line and identified two candidates with high knockout efficiency, namely sgTRAC-1 and sgB2M-1, which were subsequently utilized for knocking out target genes in primary T cells (Supplementary Fig. 4A-B). *Cas9* mRNAs and target gRNAs were co-nucleofected into activated CD8^+^ T cells twice to achieve high gene disruption efficiency (Supplementary Fig. 4C). The DNA fragments containing gRNA-targeted sites were amplified from the transduced cells, followed by the T7E1 mismatch detection assay and DNA sequencing assay to validate knockout efficiencies of *TRAC* and *B2M* genes. The agarose gel electrophoresis results revealed that multiple cleavage bands were presented within DNA from knockout cells upon T7E1 treatment (Supplementary Fig. 4D). Besides, multiple peaks appeared within DNA from knockout cells in Sanger sequencing, which indicated that CRISPR/Cas9-mediated events of NHEJ were occurred at the *TRAC* and *B2M* genomic loci (Supplementary Fig. 4E). In addition, single clonal sequence analysis revealed that indels and insertions occurred after CRISPR/Cas9-mediated recombination of *TRAC* and *B2M* loci respectively (Supplementary Fig. 4F).

**Fig. 2:**
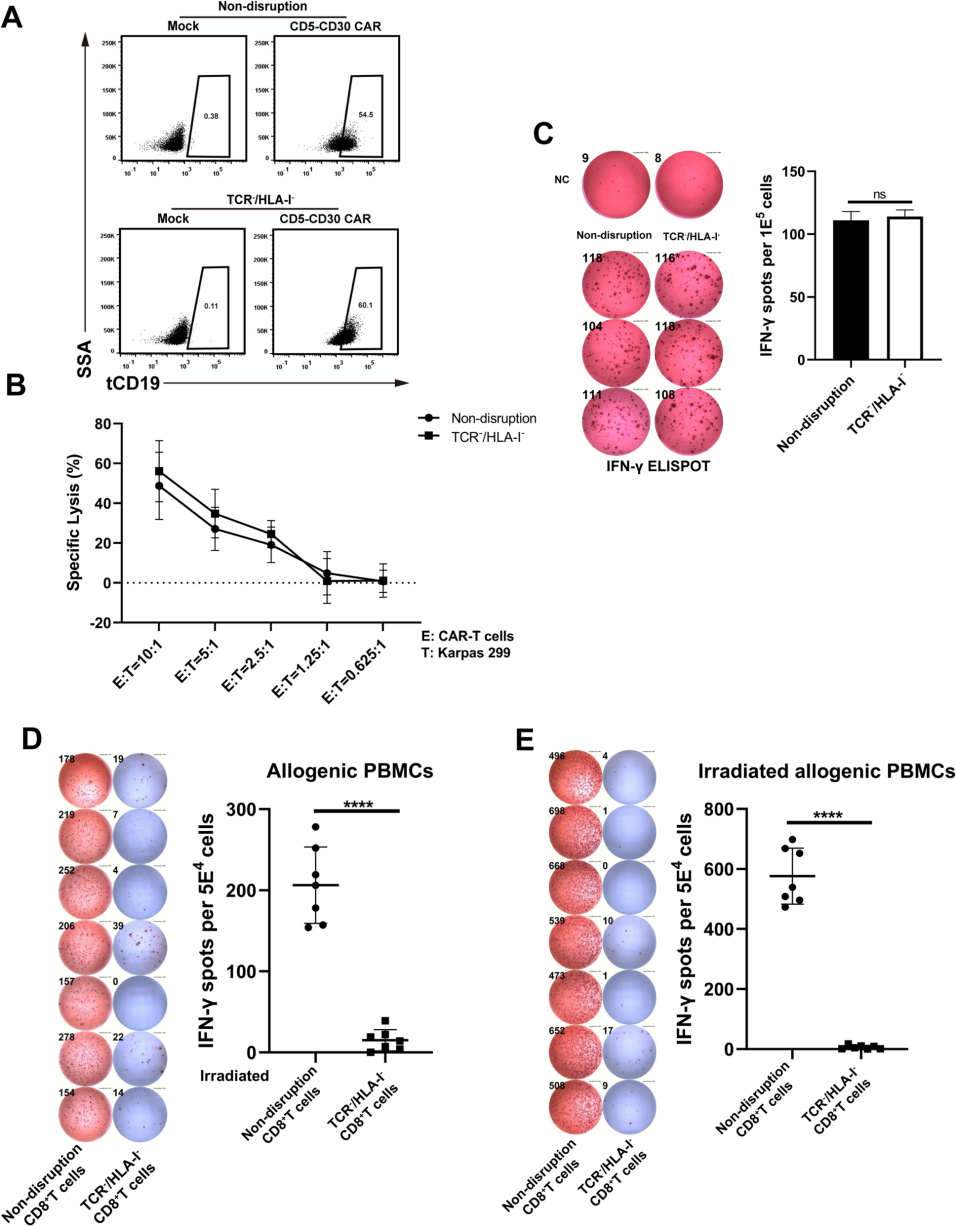
CRISPR/Cas9-mediated TCR and HLA-I knockout did not affect the function of T cells and reduced alloreactivity. **A** The expression of CD5-CD30-CAR on non-disrupted and TCR^−^/HLA-I^−^ CD8^+^ T cells. tCD19 was in-frame expressed with CD5-CD30-CAR to indicate the expression of CAR molecules. **B** The killing results of Karpas 299 target cells under different effector/target ratios. Data represented as mean ± SD. *N* = 3 independent biological replicates. **C** CD5-CD30-CAR-transduced CD8^+^ T cells derived from non-disrupted and TCR^−^/HLA-I^−^ cells were co-cultured with Karpas 299 at a ratio of 4:1 respectively. IFN-γ secretion within each group was analyzed by ELISPOT assay. Data represented as mean ± SD. *N* = 3 independent biological replicates. **D** and **E** The inhibition of alloreactivity upon TCR/HLA-I disruption was determined utilizing the IFN-γ ELISPOT assay. Allogenic PBMCs were co-cultured with irradiated gene-edited T cells (**D**). Irradiated allogenic PBMCs were also co-cultured with gene-edited T cells (**E**). Data represented as mean ± SD. *N* = 7 independent biological replicates. Statistical analysis was performed by Student’s *t*-test. **** *p* < 0.0001

To evaluate whether the simultaneous disruption of TCR and HLA-I would affect the proliferation and effector function of T cells, we enriched TCR and HLA-I double-negative (TCR^−^/HLA-I^−^) CD8^+^ T cells. No expansion difference was observed between non-disrupted and TCR^−^/HLA-I^−^ cells (Supplementary Fig. 5A). Besides, TCR^−^/HLA-I^−^ cells permanently maintained the double-negative state (Supplementary Fig. 5B). The effector function was detected after the transduction of CD5-CD30 bispecific CAR into TCR^−^/HLA-I^−^ T cells (Fig. 2A). When CAR-T cells were co-incubated with Karpas 299 cells, the killing activity and IFN-γ secretion ability of TCR^−^/HLA-I^−^ CAR-T cells were equal to those of non-disrupted T cells (Fig. B-C). These results indicated that CRISPR/Cas9-mediated TCR and HLA-I knockout did not affect the proliferation and function of T cells.

Next, the allogeneic lymphocytes co-culturing assay was used to validate whether the alloreactivity has been eliminated within TCR^−^/HLA-I^−^ T cells. The data showed that the absence of HLA-I molecules resulted in a significant reduction of alloreactivity, as evidenced by the absence of obvious IFN-γ secretion responses when allogeneic PBMCs were co-cultured with irradiated TCR^−^/HLA-I^−^ cells (Fig. 2D). Meanwhile, we did not observe significant rejection reaction of TCR^−^/HLA-I^−^ cells on allogeneic irradiated PBMCs, which was evidenced by the absence of abundant IFN-γ-secreting TCR^−^/HLA-I^−^ cells (Fig. 2E). These data demonstrated that the simultaneous deletion of both TCR and HLA-I significantly reduced alloreactivity.

### The screening and optimization of candidate T cells with high plasticity

In recent years, the culturing of immune cells within both basic researches and clinical applications has increasingly tended towards utilizing serum-free media [42]. This culturing system can significantly reduce the risk of introducing heterologous infections during cell culture, and avoid potential cell activation or inactivation caused by unknown components present in serum. Therefore, we selected five candidate media (RPMI 1640, KBM 581, X-VIVO 15, OptiVitro T-SFM and ImmunoCult-XF) to identify the medium with the most rapid expansion rate, which is vital for preparing large amounts of accessible CAR-T cells within short time. These media have commercial applications and have been utilized in research on T cells culture and expansion [43, 44]. The successful screening of optimal medium would facilitate subsequent large-scale production and clinical research. We evaluated various media to identify the OptiVitro T-SFM medium with the best amplification advantage, particularly after 12 days of culturing, making it a suitable medium for long-term culturing and expansion of T cells (Supplementary Fig. 6A).

**Fig. 3:**
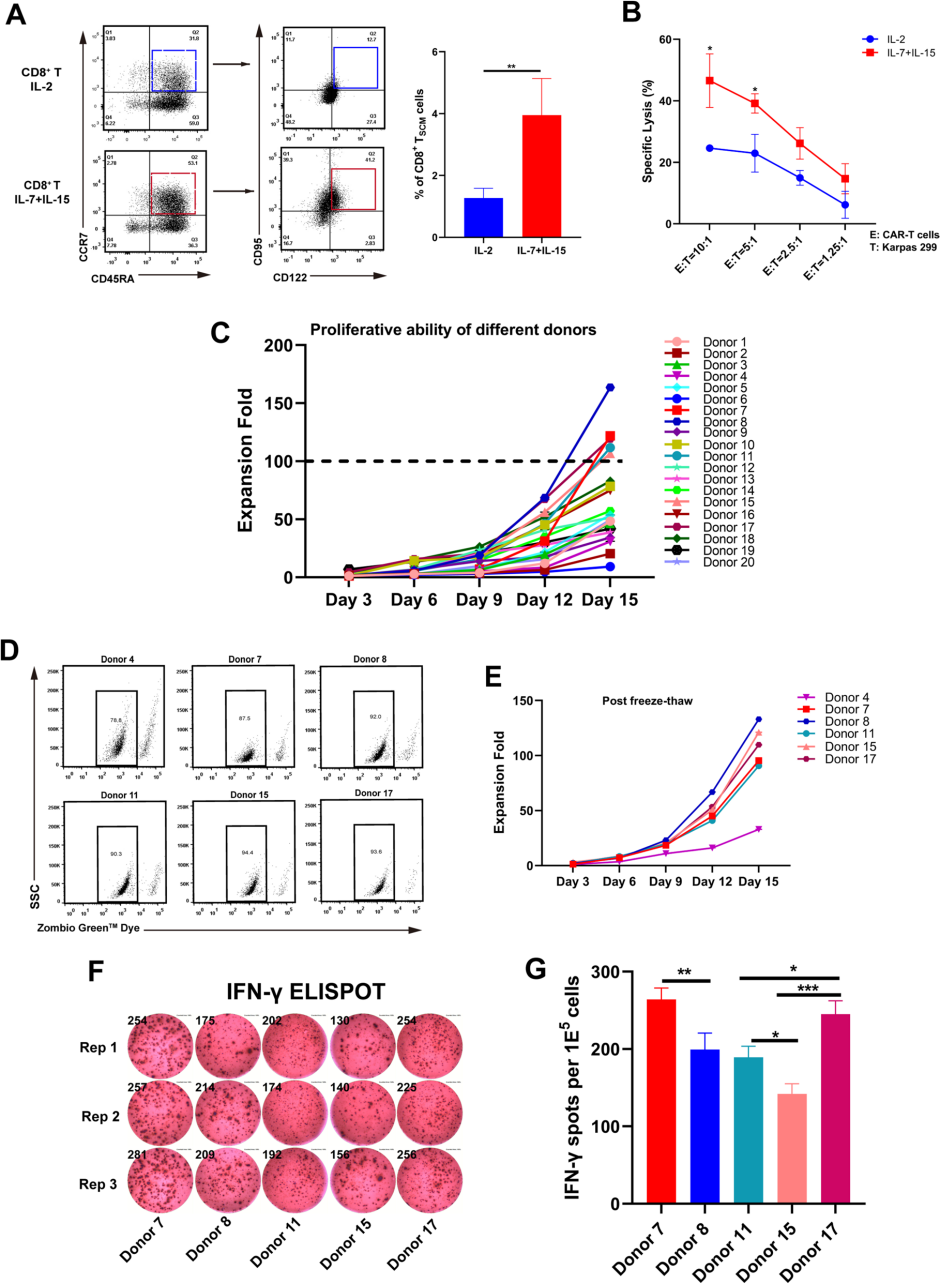
Candidate T cells were screened and obtained utilizing modified culture conditions. **A** Phenotypic analysis of T cells was performed based on the expression of CD45RA, CCR7, CD122 and CD95 after exposure to either IL-2 or a combination of IL-7 and IL-15. The percentage of T_SCM_ (CD45RA^+^ CCR7^+^ CD122^+^ CD95^+^) cells was shown. Data represented as mean ± SD. *N* = 9 independent biological replicates. **B** T cells which were induced by either IL-2 or the combination of IL-7 and IL-15 were mixed with Karpas 299 target cells to conduct the cytotoxicity assay. Data represented as mean ± SD. *N* = 3 independent biological replicates. **C** Expansion of CD8^+^ T cells derived from 20 healthy donors within OptiVitro T-SFM medium containing IL-7 and IL-15. **D** and **E** The viability and expansion of five dominant CD8^+^ T cells, along with one suboptimal CD8^+^ T cells, following cryopreservation and thawing cycles. **F** and **G** TCR^−^/HLA-I^−^ CD8^+^ T cells derived from five dominant donors were transduced with CD5-CD30 CAR and incubated with Karpas 299 cells for 18 to 24 h. IFN-γ secretion within each group was analyzed utilizing ELISPOT assays. Data in (**A**) were analyzed by Student’s *t*-test. Data in (**B**) were analyzed by two-way ANOVA with Sidak’s multiple comparisons test. Data in (**G**) were analyzed by one-way ANOVA with Tukey’s multiple comparisons test. * *p* < 0.05, ** *p* < 0.01, *** *p* < 0.00

T_SCM_ cells, a subset of memory T cells that possess self-renewal and multi-directional differentiation potential, are considered to have promising prospects for applications in adoptive immunotherapy [24, 45]. Studies have demonstrated that IL-2 can induce the over-activation of T cells and the apoptosis of activated T cells, while cytokines including IL-7, IL-15 and IL-21 tend to maintain T cells in a state of higher developmental plasticity without inducing cellular exhaustion [46–48]. T_SCM_ cells and naïve T cells share multiple phenotypic characteristics, such as CD45RA^+^, CD45RO^−^, CCR7^+^ and CD27^+^. However, T_SCM_ cells can be distinguished from naïve T cells by their higher expression of CD95 and CD122 (IL-2Rβ) [49–51]. In agreement with previous studies, our experiments demonstrated that not only more CD8^+^ T_SCM_ subsets could be induced, but also a faster expansion rate could be achieved through co-culturing with IL-7 and IL-15 cytokines after 2 days of activation by anti-CD3 and anti-CD28 antibodies (Supplementary Fig. 4B, Fig. 3A). Additionally, we transduced CD5-CD30 bispecific CAR into CD8^+^ T cells that were stimulated with these two combinations of cytokines. Karpas 299 cells were employed as target cells for killing experiments. The data demonstrated that IL-7- and IL-15-induced cells exhibited superior cytotoxicity than IL-2-induced cells (Fig. 3B). Therefore, the final culture conditions comprised by OptiVitro T-SFM medium supplemented with IL-7 and IL-15 was utilized to expand T cells in subsequent experiments.

Following the establishment of the T cells culture and expansion protocol described above, different allogeneic T cells were screened to obtain dominant T cells with a faster expansion rate and stronger effector function for constructing MU-CAR-T cells. We monitored the expansion rate of CD8^+^ T cells from 20 healthy donors, and identified the top five candidates including Donor 7, Donor 8, Donor 11, Donor 15 and Donor 17 which possessed faster expansion rates (Fig. 3C). To assess the impact of freeze-thaw on cellular viability, CD8^+^ T cells derived from five dominant donors were stored in liquid nitrogen for one week and subsequently thawed for re-culturing. After cryopreservation and thawing, cell viabilities of these candidates were approximately 90%, with no significant impact on their proliferative capacity. Furthermore, we tested a suboptimal donor to demonstrate that the freeze-thaw has minimal impact on the viability and amplification of T cells (Fig. 3D-E). Then, TCR and HLA-I molecules of these T cells were knocked out, followed by double-negative enrichment (Supplementary Fig. 7). Subsequently, the effector functions of these TCR^−^/HLA-I^−^ cells were validated by IFN-γ ELISPOT assay. These enriched T cells were transduced with CD5-CD30 CAR and co-incubated with Karpas 299 target cells. The data showed that Donor 7 and Donor 17 secreted higher levels of cytotoxic cytokines when stimulated by target cells compared to those in other groups (Fig. 3F-G). These data revealed that CD8^+^ T cells derived from Donor 7 and Donor 17 exhibited superior expansion capacities, better tolerance for freeze-thaw, and accompanied by stronger effector function. Therefore, these two donors-derived CD8^+^ T cells were designated as dominant candidates.

#### Generation and functional validation of MU-CAR-T cells

**Fig. 4:**
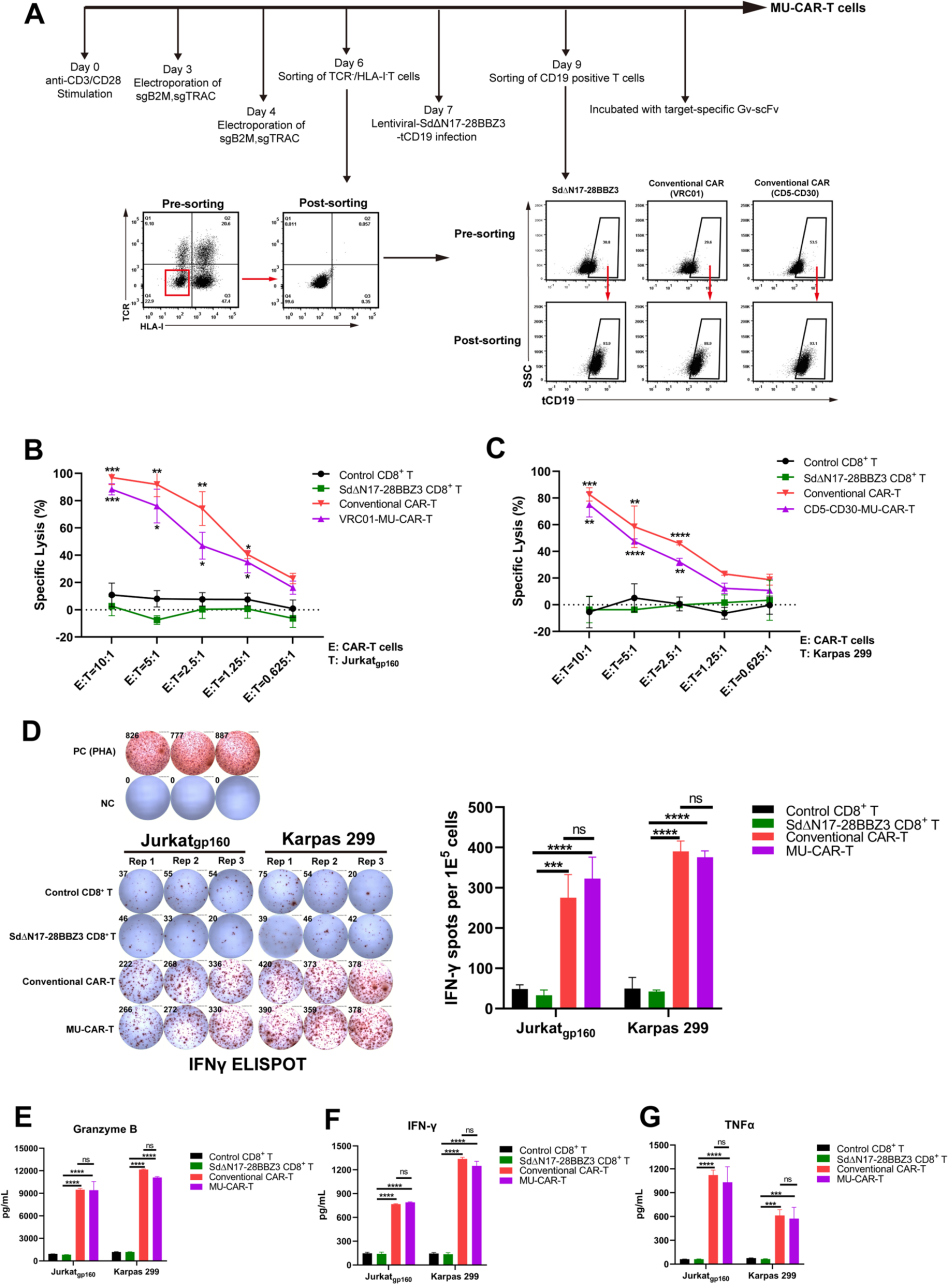
Generation and functional validation of MU-CAR-T cells. **A** Flowchart illustrating the protocol for generating MU-CAR-T cells. Briefly, α-CD3/α-CD28-activated CD8^+^ T cells were electroporated with *Cas9* mRNA and sgRNAs targeting *B2M* and *TRAC* on Day 3 and Day 4. On Day 6, TCR^−^/HLA-I^−^ T cells were enriched. Then, enriched T cells were transduced with SdΔN17-28BBZ3-tCD19 through lentiviral infection on Day 7. CD19-positive selection was performed on Day 9. After incubation with the specific Gv-scFv, the resulting products were identified as MU-CAR-T cells. **B** and **C** The cytotoxicity of MU-CAR-T cells against Jurkat_gp160_ and Karpas 299 target cells was observed upon being conjugated with VRC01 scFv (**B**) and CD5-CD30 scFvs (**C**) respectively. Data represented as mean ± SD. *N* = 3 independent biological replicates. **D** Control CD8^+^ T, SdΔN17-28BBZ3-expressing CD8^+^ T, conventional CAR-T and MU-CAR-T cells were co-cultured with target cells at a 4:1 ratio respectively. IFN-γ secretion was detected utilizing ELISPOT assay. PHA-stimulated effector cells were treated as the positive control (PC), and effector cells-only group was treated as the negative control (NC). Data represented as mean ± SD. *N* = 3 independent biological replicates. (**E**, **F** and **G**) The production of cytokines including Granzyme B (**E**), IFN-γ (**F**) and TNFα (**G**) was assessed upon co-culturing of control CD8^+^ T cells and CAR-T cells with different target cells at the ratio of 4:1. Data represented as mean ± SD. *N* = 3 independent biological replicates. Data in (**B**) and (**C**) were analyzed by two-way ANOVA with Tukey’s multiple comparisons test. Data in (**D**-**G**) were analyzed by one-way ANOVA with Tukey’s multiple comparisons test. * *p* < 0.05, ** *p* < 0.01, *** *p* < 0.001, **** *p* < 0.0001

The Sd/Gv may have potential immunogenicity due to their bacterial origin. Therefore, to reduce the immunogenicity of Sd/Gv, we constructed a series of truncated variants of Sd while preserving its function. Ultimately, we obtained the optimal variant SdΔN17 which was truncated 17 amino acids from the N-terminus. We used the BepiPred-2.0 prediction tool to assess the immunogenicity of Sd and SdΔN17, and the results revealed that SdΔN17 has fewer linear B-cell epitopes compared to Sd (Supplementary Figs. 8 and 9). More importantly, we have demonstrated that mice immunized with Gv/Sd exhibit lower antibody titers against the Gv/Sd protein itself, indicating its inherent low immunogenicity. Additionally, the pre-existing antibodies against Gv/Sd protein were nearly not detected in healthy individuals, suggesting that this construct can be utilized for clinical applications [26]. Therefore, the optimal variant SdΔN17 was selected for subsequent in vitro and in vivo efficacy studies. Based on results of the above experiments, we utilized dominant T cells derived from Donor 17 to generate MU-CAR-T cells. Activated T cells underwent two rounds of electroporation to knock out *TCR* and *HLA-I* genes, followed by the enrichment of TCR^−^/HLA-I^−^ T cells. Subsequently, enriched T cells were transduced with SdΔN17-28BBZ3-tCD19, followed by positive sorting for CD19-expressing cells. After incubation with the specific Gv-scFv, resulting products were identified as MU-CAR-T cells (Fig. 4A). Two specific antigen-recognition domains, namely VRC01 scFv and CD5-CD30 scFvs, were utilized to validate the efficacy of MU-CAR-T cells as described above. Meanwhile, conventional CAR-T cells including VRC01-CAR-T cells and CD5-CD30-CAR-T cells were generated as positive controls. These cells were also enriched for CD19-positive population to obtain T cells with high levels of CAR expression (Fig. 4A). The cytotoxic potency of MU-CAR-T cells was evaluated by detecting the release of LDH. Our results showed that MU-CAR-T cells exhibited similar killing effects to conventional CAR-T cells on both Jurkat_gp160_ and Karpas 299 target cells (Fig. 4B-C). Moreover, the cytotoxicity of MU-CAR-T cells was comparable to that of conventional CAR-T cells at an effector-to-target ratio of 10:1. We next explored the functional properties of MU-CAR-T cells in vitro. The IFN-γ ELISPOT assay results showed that the numbers of IFN-γ-positive spots formed in the MU-CAR-T group were significantly higher than those in control and SDΔN17-28BBZ3 groups upon stimulated with target cells (Fig. 4D). While there were no significant differences between MU-CAR-T groups and conventional CAR-T groups. Further ELISA assays against cytotoxic molecules or cytokines showed that, like conventional CAR-T cells, MU-CAR-T cells also secreted significantly higher concentrations of Granzyme B, IFN-γ and TNFα than those in mock and SDΔN17-28BBZ3 groups (Fig. 4E-G). These results indicated that the activation of cellular signaling and killing of target cells depended on the presence of specific scFv, and the effector functions of generated MU-CAR-T cells were comparable to those of conventional CAR-T cells.

### Suppression of HIV-1 latency rebound by MU-CAR-T cells

**Fig. 5:**
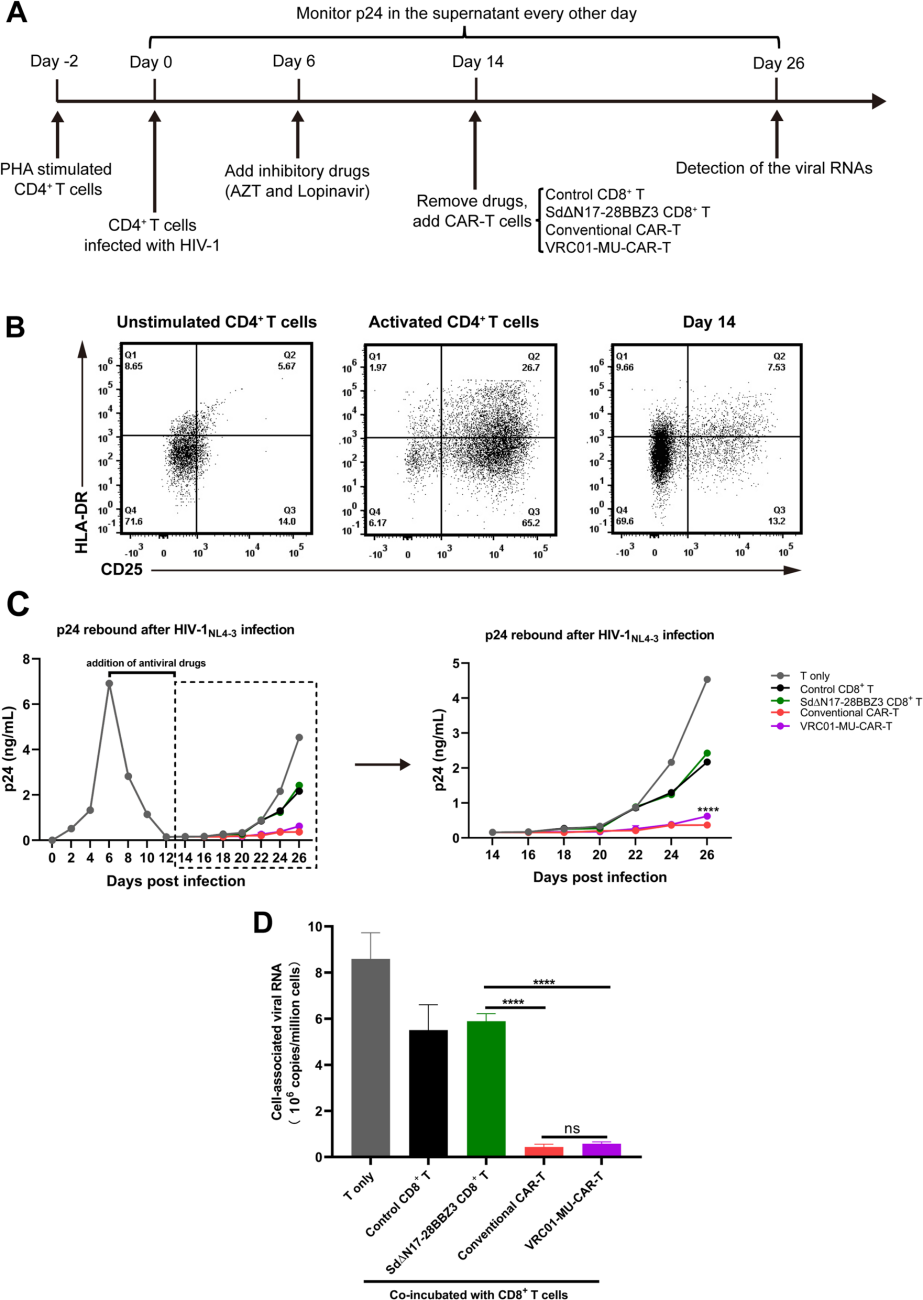
MU-CAR-T cells efficiently suppressed HIV-1 rebound after the withdrawal of antiviral treatment in vitro. **A** Flow chart of the experimental design. PHA-activated CD4^+^ T cells were infected with wild-type HIV-1_NL4 − 3_. On Day 6, inhibitory drugs including AZT and Lopinavir were added to inhibit viral replication. On Day 14, anti-HIV-1 drugs were withdrawn, and infected CD4^+^ T cells were co-cultured with various groups of T cells including control CD8^+^ T cells, SdΔN17-28BBZ3-expressing CD8^+^ T cells, conventional CAR-T and VRC01-MU-CAR-T cells. On Day 26, infected CD4^+^ T cells were harvested to detect viral RNAs. HIV-1 p24 proteins within the supernatant were monitored every 2 days. **B** The expression of activation markers including CD25 and HLA-DR was analyzed on unstimulated and activated CD4^+^ T cells, as well as CD4^+^ T cells on Day 14 post infection. The percentage of cells was indicated in each quadrant. **C** Culture supernatants were tested for the presence of p24 utilizing ELISA assay every 2 days, which represented the expression and rebound of HIV-1. Data represented as mean ± SD. *N* = 3 independent biological replicates. **D** Cells from each group were harvested on Day 26, and cell-associated HIV-1 RNAs were quantified utilizing real-time RT-qPCR. Data represented as mean ± SD. *N* = 3 independent biological replicates. Data in (**C**) were analyzed by one-way ANOVA with Tukey’s multiple comparisons test. Data in (**D**) were analyzed by two-way ANOVA with Tukey’s multiple comparisons test. **** *p* < 0.0001

The inhibitory effect of MU-CAR-T was further validated through in vitro wild-type HIV-1 infection and drug withdrawal model. We attempted to simulate the process of viral rebound in vivo by withdrawing antiviral treatment. PBMCs were obtained from healthy donors and proceeded to sort CD4^+^ T cells which were utilized as target cells for HIV-1 infection. Six days post infection of CD4^+^ T cells with wild-type HIV-1_NL4 − 3_, antiretroviral compounds including AZT and lopinavir were added to the culture medium to suppress viral replication and prevent further spread of infection (Fig. 5A). Concurrently, infected cells were sustained in the presence of a very low concentration of IL-2. After approximately 8 to 10 days post drug treatment, the viral yield significantly decreased to the lower limit of p24 detection, and infected CD4^+^ T cells were close to a quiescent state (Fig. 5B). Subsequently, we discontinued the administration of anti-HIV-1 drugs and introduced VRC01-CAR-T cells, VRC01-MU-CAR-T cells, SdΔN17-28BBZ3-expressing CD8^+^ T cells or control CD8^+^ T cells respectively. In the absence of CD8^+^ T cell co-culturing, HIV-1 rebounded rapidly in CD4^+^ T cells, while both control and SdΔN17-28BBZ3 groups exhibited only limited suppression of viral rebound (Fig. 5C). Importantly, VRC01-MU-CAR-T cells showed significant and persistent suppression of HIV-1 rebound, the effect of which was similar to that of conventional VRC01-CAR-T cells (Fig. 5C). Meanwhile, the data also showed a significant reduction in the level of cell-associated viral RNAs within HIV-1-infected CD4^+^ T cells (Fig. 5D). These results suggested that VRC01-MU-CAR-T cells exhibited comparable efficacy to conventional VRC01-CAR-T cells in eliminating wild-type HIV-1-producing cells.

### Growth suppression of tumor in vivo by MU-CAR-T cells

**Fig. 6:**
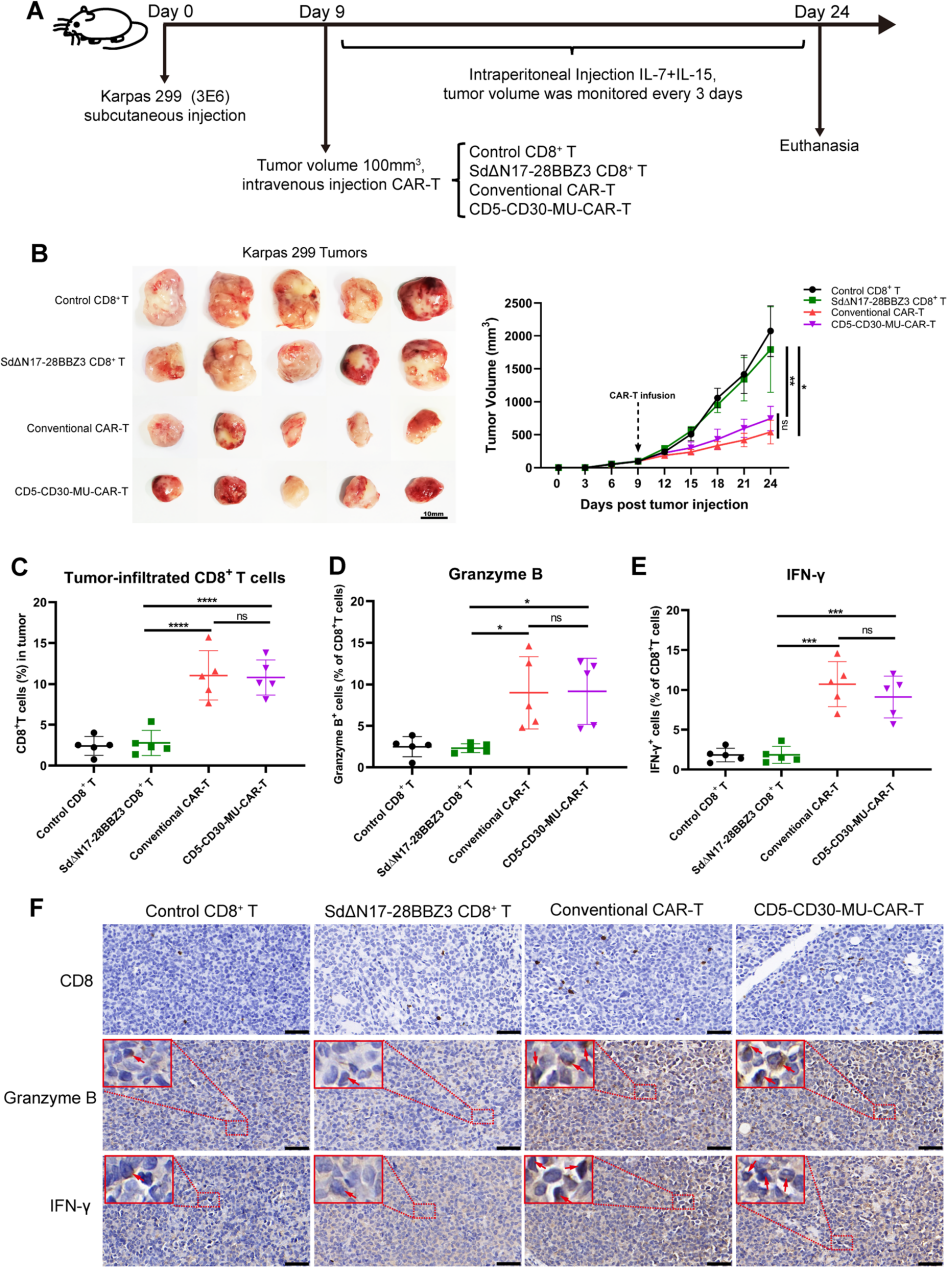
The effectiveness of MU-CAR-T cells within tumor-bearing mice in vivo*. ***A** Flow chart of the experimental design. NCG mice were subcutaneously injected with Karpas 299 lymphoma cells. On Day 9, when the tumor volume reached approximately 100 mm^3^, control CD8^+^ T, SdΔN17-28BBZ3-expressing CD8^+^ T, conventional CAR-T and CD5-CD30-MU-CAR-T cells were intravenously injected into mice respectively, followed by the intraperitoneal injection of IL-7 and IL-15 every 3 days. On Day 24, mice were euthanized and their tumors were collected for subsequent experiments. **B** Mice from each group were monitored for tumor growth every 3 days. These mice were euthanized at two weeks after infusion of CAR-T cells, and their tumors were collected. Scale bar represented 10 mm. **C**, **D**, and **E**) Mononuclear cells were isolated from collected tumors. Subsequently, the proportion of infiltrating T cells (**C**) as well as their capabilities of Granzyme B (**D**) and IFN-γ (E) secretion were evaluated utilizing flow cytometry analysis. **F** IHC staining results of CD8^+^ T cells, Granzyme B cytotoxic molecules and IFN-γ cytokines were acquired within tumors derived from various groups. Scale bars represented 50 μm. Data represented as mean ± SD. *N* = 5 independent biological replicates. Data in (**B**) were analyzed by two-way ANOVA with Tukey’s multiple comparisons test. Data in (**C**-**E**) were analyzed by one-way ANOVA with Tukey’s multiple comparisons test. * *p* < 0.05, ** *p* < 0.01, *** *p* < 0.001, **** *p* < 0.0001

The in vivo efficacy of MU-CAR-T cells was also evaluated in xenograft tumor model utilizing NCG mice. These mice were inoculated with Karpas 299 cells via subcutaneous injection on the right side of their backs. Mice were monitored for tumor growth every 3 days. When the tumor volume reached approximately 100 mm^3^, CD5-CD30-MU-CAR-T cells, conventional CD5-CD30-CAR-T cells, SdΔN17-28BBZ3-expressing CD8^+^ T cells or control CD8^+^ T cells were adoptively transferred into NCG mice via tail vein injection respectively. Besides, the stimulation of IL-7 and IL-15 leads to the induction of highly plastic T cells. Therefore, IL-7 and IL-15 were administered intraperitoneally at a dose of 2 µg / 200 uL every 3 days after infusion to support the maintenance and expansion of CAR-T cells (Fig. 6A). These T cells-treated mice were euthanized at two weeks after infusion of CAR-T cells, and their tumors were collected for subsequent experiments. The data showed that CD5-CD30 MU-CAR-T cells significantly inhibited tumor growth compared with control and SdΔN17-28BBZ3 groups (Fig. 6B). Furthermore, the anti-tumor efficacy of CD5-CD30 MU-CAR-T cells was comparable to that of conventional CD5-CD30 CAR-T cells.

Next, we further explored the tumor-infiltration capabilities of CD5-CD30-MU-CAR-T cells. Collected tumors were divided into two sections, one for isolation of tumor-infiltrating mononuclear cells and the other for the preparation of immunohistochemical (IHC) staining. We dissected tumor tissues from each mouse and extracted an equivalent volume of tissue for subsequent isolation of infiltrating mononuclear cells. Then, the proportion of CD8^+^ T cells in infiltrating mononuclear cells was determined by flow cytometry analysis. The data showed that higher percentages of tumor-infiltrating CD8^+^ T cells were presented in both the CD5-CD30-MU-CAR-T group and the conventional CAR-T group, compared with those in control group or SdΔN17-28BBZ3 group (Fig. 6C). Concurrently, intracellular cytotoxic molecules or cytokines were stained to assess the functionality of CAR-T cells in the tumor by measuring their production of Granzyme B and IFN-γ. These findings also showed that infiltrated CD5-CD30-MU-CAR-T cells exhibited robust production of Granzyme B and IFN-γ, which was similar to conventional CAR-T cells (Fig. 6D-E). In addition, IHC results showed that both CD5-CD30-MU-CAR-T and conventional CAR-T groups exhibited higher infiltration of CD8^+^ T cells and more robust capacity to secrete Granzyme B and IFN-γ, which were consistent with the flow cytometry data mentioned above (Fig. 6F). Taken together, MU-CAR-T cells effectively inhibited tumor growth in vivo and exhibited robust ability to infiltrate tumor and secrete cytokines.

Taken together, MU-CAR-T cells were successfully constructed based on the Sd/Gv system. T cells with the double knockout of TCR and HLA-I significantly reduced the alloreactivity. Moreover, the efficacy of MU-CAR-T cells had been demonstrated for their therapeutic effects on anti-infection and anti-tumor models in vitro and in vivo. Importantly, our construction strategy enhanced the modularization of universal CAR-T (UCAR-T) cells and facilitated the implementation of quality control measurements for UCAR-T products (Supplementary Fig. 10). The process of producing allogeneic CAR-T cells began with isolating and enriching T cells from healthy individuals. Plasticity T cells were obtained by screening and optimizing from multiple donors. The CRISPR/Cas9 gene editing technology was used to eliminate TCR and HLA-I on T cells, resulting in the reduction of alloreactivity. Then the SdΔN17-28BBZ3 moiety was transduced into TCR^−^/HLA-I^−^ T cells. Subsequently, these cells were amplified, sub-packaged and stored in liquid nitrogen for future manipulation. MU-CAR-T cells were generated through covalent attachment with various Gv-tagged scFvs on SdΔN17-28BBZ3-expressing T cells, and applied to various patients suffering from different diseases. The established MU-CAR-T cells could be stored, transported and administrated to patients expeditiously, which significantly shortened the time to prepare autologous or allogeneic CAR-T cells without further re-editing.

## Discussion

The conventional structure of CAR primarily comprises the extracellular single-chain variable region, the hinge or spacer region, the transmembrane domain and the intracellular signaling domain [52, 53]. With the development of molecular biology and tumor immunology, the design of CAR structures has gradually evolved from the traditional single target to a new generation of CAR with multi-targets, adjustability and controllability [54–56]. These advancements have led to enhanced safety profiles and superior anti-tumor effects. The adapter-mediated universal CAR-T cells construction is an emerging technology that effectively overcomes the design and therapeutic limitations of conventional CAR-T cells technique. The targeting ability of conventional CAR-T cells is limited to a single antigen, rendering the therapy susceptible to relapse caused by downregulation or deletion of the targeted antigen [57, 58]. Many cases of antigen-negative relapse resulting from tumor antigen evasion following CD19 CAR-T therapy have been documented in clinical trials [59, 60]. Engineering CAR-T cells targeting multiple tumor-associated antigens (TAA) represents a promising strategy to overcome tumors immune evasion. However, the practicality of this approach is limited by both the technical requirements and costs involved in generating CARs that target individual antigens for each patient [61]. Therefore, the establishment of a platform for generating universally redirected T cells that can virtually target any antigen from any donor will be particularly important for the widespread application of CAR-T therapy.

Here, we propose a construction protocol for generating novel MU-CAR-T cells. The functional activation of MU-CAR-T cells is exclusively dependent on the presence of scFv, and replacing the Gv-labeled scFv that enables redirection of T cells without requiring redesign and time-consuming remanufacturing. Our findings demonstrate the feasibility of this protocol, as generated MU-CAR-T cells are successfully validated for their target recognition and cytotoxicity mediated by VRC01-scFv or CD5-CD30 scFvs for latent HIV-1-infected cells or T cell lymphoma cells respectively. Furthermore, the *tCD19* gene is located downstream of the CAR structure, serving as both a screening marker and a “suicide gene”. In cases of adverse effects during MU-CAR-T cells therapy, the administration of CD19-targeted pharmaceutical agent such as Inebilizumab can effectively eliminate MU-CAR-T cells and mitigate their toxic side effects [62].

To prevent GVHD and host-versus-graft reactions, it is necessary to eliminate TCR and HLA-I molecules. Torikai et al. reported for the first time that CD19 CAR-T cells were edited by the *ZFN* gene to eliminate the expression of endogenous αβ TCR, thus preventing graft-versus-host reaction without affecting the effector function of CAR, which became the initial model of UCAR-T [63]. Ren et al. used CRISPR/Cas9 technology to knock out endogenous *TCR*, *B2M* and *PD1* genes simultaneously, resulting in allogeneic CAR-T cells lacking TCR, HLA-I and PD1 molecules, which decreased their allogenic activity and avoided graft-versus-host disease, as well as enhanced antitumor efficacy [64]. . Our technique reported here coalesces the above reported strategies, which shows that the expansion and killing abilities of T cells are not affected by the knockout of the *TCR* and *HLA-I* genes. While the double knockout strategy significantly reduces the transplantation rejection.

Additionally, allogeneic T lymphocytes are screened, optimized and edited to establish a multi-potent, long-term maintained and high-quality cell bank in this study. The viability and function of T cells vary among individuals due to factors including genetics, lifestyle habits, immune system state and other potential variables. IL-7 maintains T cells in a less differentiated state, while IL-15 plays a pivotal role in generating and maintaining memory cells [65, 66]. T_SCM_ cells are considered the optimal candidates for maintaining a sustained response in vivo following adoptive T cells transplantation due to their superior effector function and high proliferative capacity [67, 68]. Therefore, we screen T cells from various donors to obtain dominant T cells and optimize culture conditions to obtain higher proportion of T_SCM_ cells.

The combination form of scFv attached to T cells via SdΔN17/Gv system resembles that of the antibody-drug conjugate (ADC). ADC is a targeted biological agent comprised of monoclonal antibody conjugated to cytotoxic agent (payload) via a chemical linker, enabling precise and efficient eradication of cancer cells [69, 70]. It has become a promising study field in developing anti-tumor drugs. However, payloads required for ADC design often have high toxicity, which can lead to serious side effects and drug resistance, and the linker has limited stability in circulation and is susceptible to non-specific cleavage, resulting in the release of cytotoxic payloads in normal tissues [71, 72]. Conversely, MU-CAR-T cells, as “antibody-cell conjugate” drugs, directly induce the death of target cells through the physiological functions of cytotoxic T lymphocytes rather than relying on drug toxicity. The potential side effects including hepatotoxicity and nephrotoxicity are avoided. Both the MU-CAR-T and ADC are “off-the-shelf” drugs, meaning they can be manufactured in advance on a large scale and used for patients when needed, which providing a wider range of treatment options for patients with diverse conditions.

Despite the significant advantages of MU-CAR-T cells over traditional CAR-T cells in terms of cell quality control, preparation time, large-scale production and application range, they also bring new challenges. Firstly, modular CAR relies on adding exogenous components Sd/Gv. Although the Sd domain has been optimized, infusion of non-endogenous elements may still cause immunogenicity. Its effect on patients remains untested. Secondly, to construct universal CAR-T cells, it is necessary to eliminate TCR and HLA-I molecules to avoid alloreactivity. While disabling HLA-I molecules on CAR-T cells can effectively reduce rejection reactions, HLA-I molecules are also the main inhibitory receptors for NK cells. Knocking out HLA-I molecules may lead to activation of host NK cells. Therefore, researchers generated engineered T cells by precisely inserting CARs and HLA-E at the TRAC and B2M loci of primary T cells, resulting in a disruption of the TCRαβ and HLA-ABC. These genetically modified CAR-T cells express both the CAR construct and the NK inhibitory molecule HLA-E, thus, they possess more immune evasion properties against alloreactive T cells and NK cells [73]. Our strategy to bring the CAR-T moiety into the chromosomal DNA was not through CRISPR-mediated locus-specific knock-in. In such a situation, we did not adapt a strategy to knock in the HLA-E gene, which indeed exhibited certain limitations. Thirdly, the effector function of MU-CAR-T cells is influenced by many aspects including the amount of proteins covalently loaded on T cells, the stability and persistence of proteins in vivo, and the attenuation of targeting caused by receptor internalization. Therefore, further exploration is needed for MU-CAR-T therapy to control its efficacy and side effects more precisely.

## Conclusions

This study provide a novel modular universal CAR-T cells generation platform based on the Sd/Gv system. Binding different scFvs can recognize various antigens. Targeting multiple TAAs simultaneously or sequentially can help prevent antigenic escape. In addition, it may allow for the control of T cells response function or reduction of side effects by managing the dosage of targeted ligands. The ultimate objective of developing MU-CAR-T cells therapy is to establish it as a “living drug” that can be efficiently and economically deployed in clinical settings for the treatment of oncological, autoimmune, and infectious diseases such as AIDS and COVID-19.

### Supplementary Information

**Additional file 1: Figure S1.** Cartoon schematic of the modular CAR and the map of corresponding lentivirus vector. **Figure S2.** The structural prediction of Gv-VRC01 scFv and Gv-CD5-CD30 scFvs by AlphaFold. **Figure S3.** The cells expressing Sd-28BBZ3 were capable of covalently conjugating with the protein Gv-GFP. **Figure S4.** Screening and validation of sgRNAs targeting TCR and HLA-I. **Figure S5.** Proliferation evaluation of electroporated T cells. **Figure S6.** Screening of culture media and cytokines. **Figure S7.** Five dominant CD8^+^ T cells which were depleted of *TRAC* and *B2M. ***Figure S8.** The optimization of Sd without affecting conjugation capability to Gv. **Figure S9.** Validation of the conjugation efficiency of optimized Sd variants. **Figure S10.** Manufacturing of MU-CAR-T cells. **Table S1. **The sequences of related constructs.

**Additional file 2.** Primary data.

## Data Availability

All data generated or analysed during this study are included in this manuscript and its supplementary information files.

## Abbreviations

CAR-T: Chimeric antigen receptor-T
U-CAR-T: Universal CAR-T
MU-CAR-T: Modular universal CAR-T
T_SCM_: Stem cell-like memory T
TCR: T cell receptor
HLA-I: Class I human leukocyte antigen
HIV-1: Human immunodeficiency virus type 1
HBV: Hepatitis B virus
HCV: Hepatitis C virus
scFvs: Single-chain variable fragments
Sd/Gv: SDCatcher/GVoptiTag
GVHD: Graft-versus-host disease
bnAb: Broadly neutralizing antibody
ALCL: Anaplastic large cell lymphoma
MHC: Human major histocompatibility complex
PBMCs: Peripheral blood mononuclear cells
TAAs: Multiple tumor-associated antigens
ADC: Antibody-drug conjugate
