# Supplementary material for: The construction of modular universal chimeric antigen receptor T (MU-CAR-T) cells by covalent linkage of allogeneic T cells and various antibody fragments
Source: Mol Cancer. 2024 Mar 11;23:53. doi: 10.1186/s12943-024-01938-8 (PMC10926606; doi:10.1186/s12943-024-01938-8)
Supplement: Supplementary file 1 — Additional file 1: Figure S1. Cartoon schematic of the modular CAR and the map of corresponding lentivirus vector. Figure S2. The structural prediction of Gv-VRC01 scFv and Gv-CD5-CD30 scFvs by AlphaFold. Figure S3. The cells expressing Sd-28BBZ3 were capable of covalently conjugating with the protein Gv-GFP. Figure S4. Screening and validation of sgRNAs targeting TCR and HLA-I. Figure S5. Proliferation evaluation of electroporated T cells. Figure S6. Screening of culture media and cytokines. Figure S7. Five dominant CD8+ T cells which were depleted of TRAC and B2M. Figure S8. The optimization of Sd without affecting conjugation capability to Gv. Figure S9. Validation of the conjugation efficiency of optimized Sd variants. Figure S10. Manufacturing of MU-CAR-T cells. Table S1. The sequences of related constructs. [file 12943_2024_1938_MOESM1_ESM.docx]

Supplemental Material

**Figure S1**

**
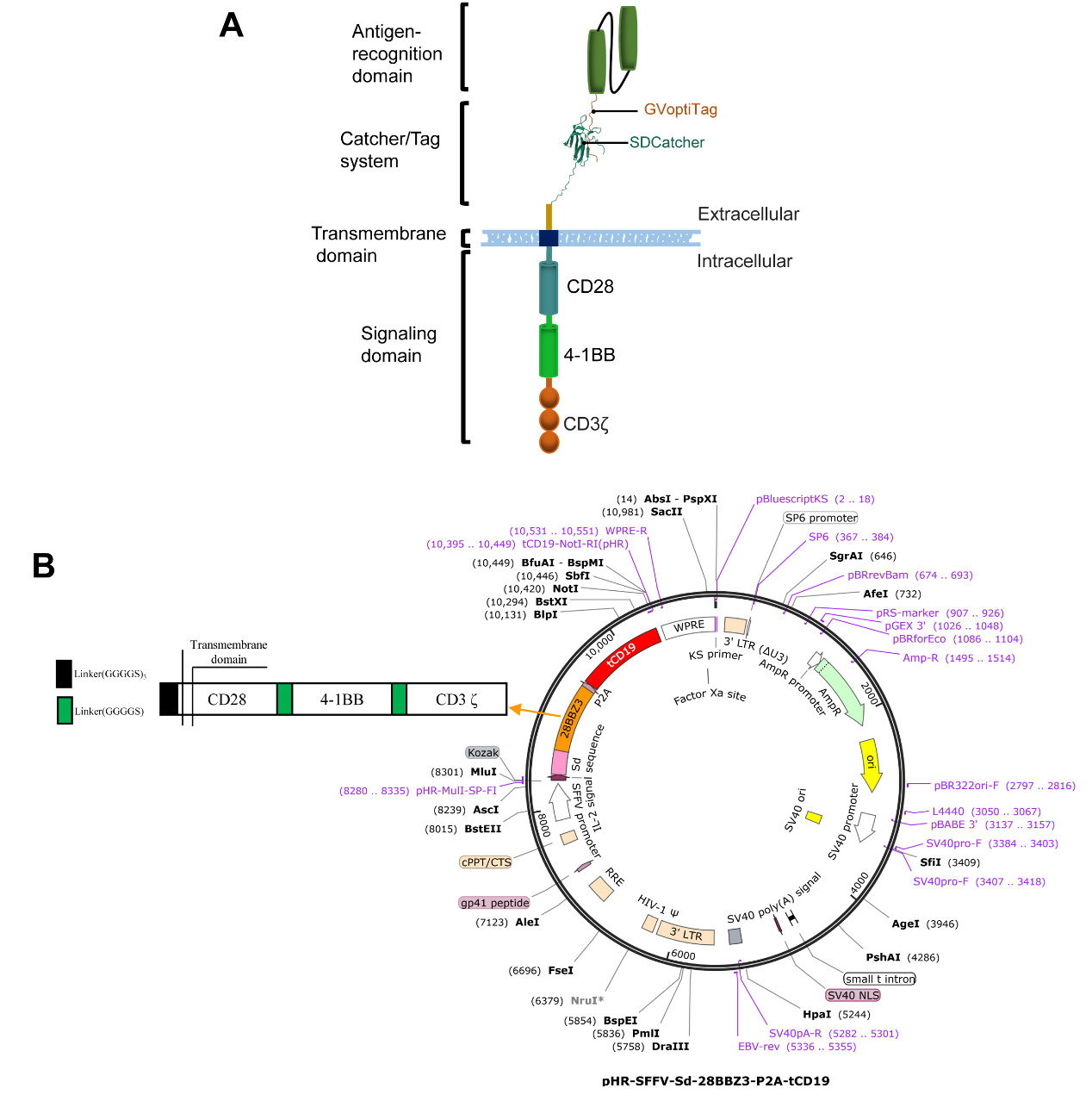
**

**Figure S1. Cartoon** **schematic of the modular CAR and the map of corresponding lentivirus vector.**

(A) The schematic of the modular CAR based on the Sd/Gv system, which containing Gv-tagged antigen-recognition domain (Gv-scFv) and Sd-tagged transmembrane and intracellular signaling domains (Sd-28BBZ3). Sd and Gv formed isopeptide bond which covalently connected two separate parts. (B) The gene fragment encoding the Sd-28BBZ3-PA2-tCD19 was cloned into the pHR vector via the MluI and NotI cloning sites. The 28BBZ3 consisted of transmembrane domain, CD28, 4-1BB and CD3ζ, which were linked by the insert linker (GGGGS)_3_ or (GGGGS) between each signaling domain.

**Figure S2**


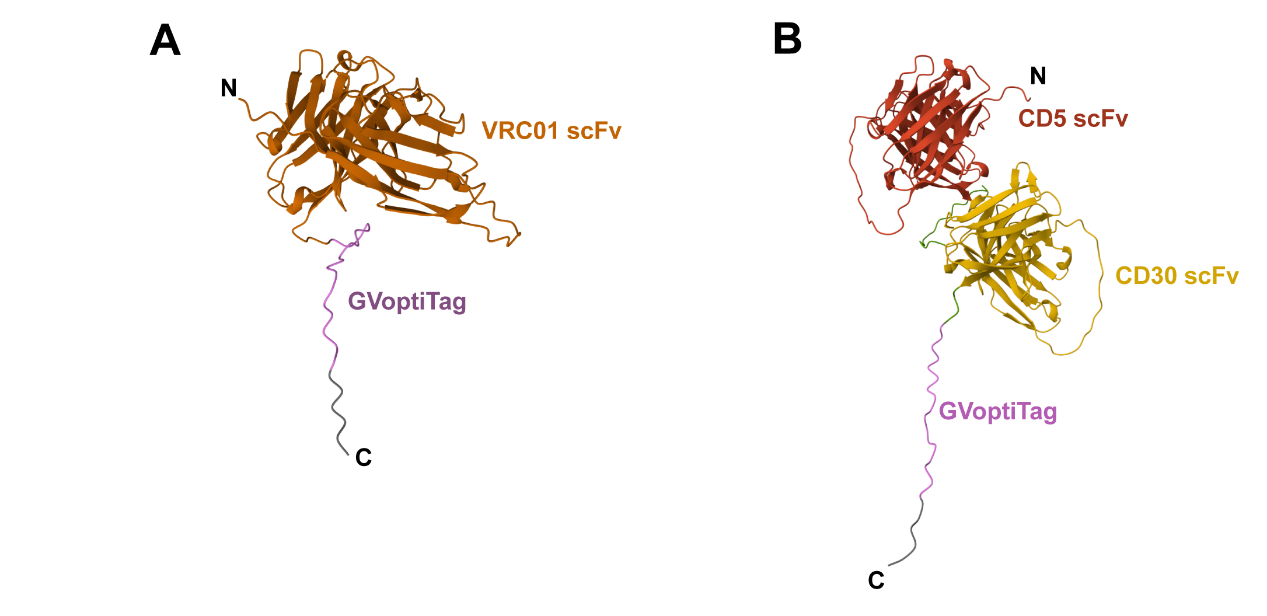


**Figure S2.** **The structural prediction of Gv-VRC01 scFv and Gv-CD5-CD30 scFvs by AlphaFold.**

(A) Gv-VRC01 scFv. (B) Gv-CD5-CD30 scFvs.

**Figure S3**

**
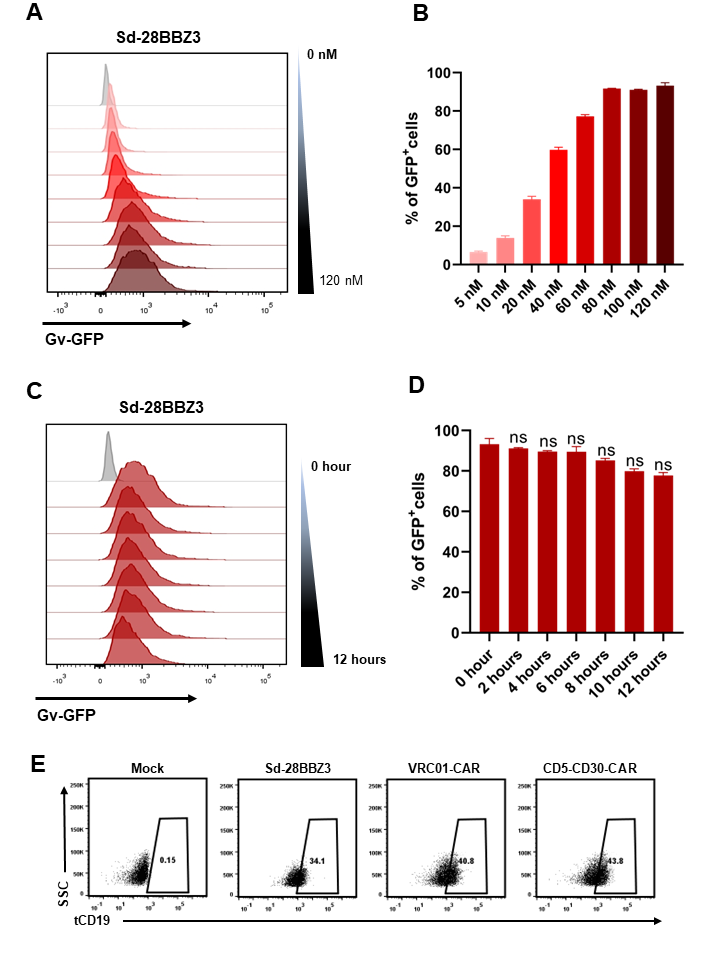
**

**Figure S3.** **The cells expressing Sd-28BBZ3 were capable of covalently conjugating with the protein Gv-GFP.**

(A) Sd-28BBZ3-expressing CD8^+^ T cells were incubated with various amounts of Gv-GFP for 12 hours at 37 ℃. The successful conjugation of Gv-GFP on Sd-28BBZ3-expressing cells was evaluated by flow cytometry. (B) Histogram showed the percentage of GFP-positive cells. (C) About 100 nM Gv-GFP proteins were co-incubated with Sd-28BBZ3-expressing cells. The conjugation of Gv-GFP onto target cells was monitored every 2 hours. (D) Histogram showed the percentage of GFP-positive cells. Data represented as mean ± SD. Data were analyzed by one-way ANOVA with Tukey’s multiple comparisons test. ns, no significance. (E) The infection efficiencies of Sd-28BBZ3, VRC01-CAR and CD5-CD30-CAR were detected using flow cytometry.

**Figure S4**

**
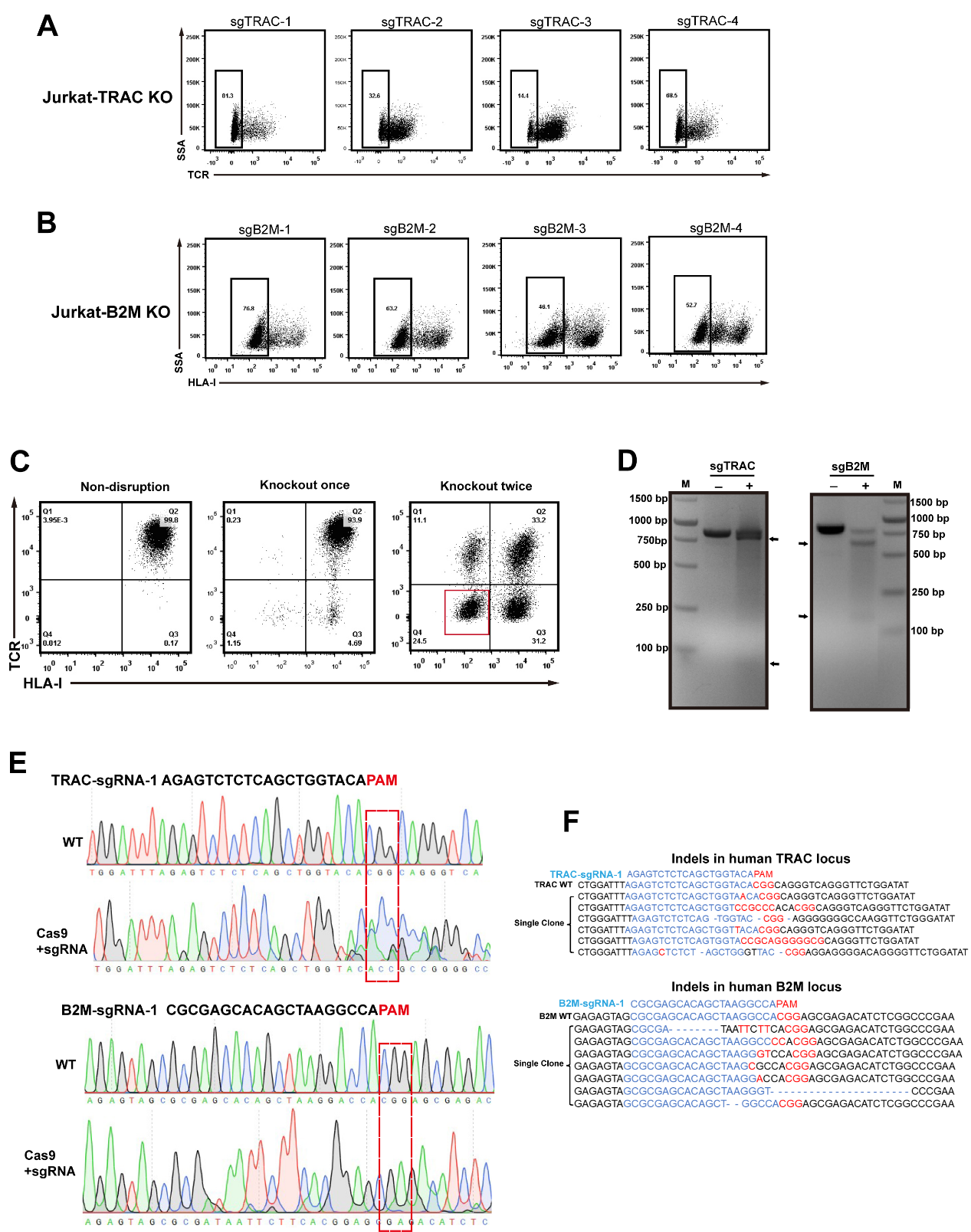
**

**Figure S4.** **Screening and validation of sgRNAs targeting TCR and HLA-I.**

(A-B) The sgRNAs targeting *TRAC* (A) and *B2M* (B) genes were screened in the Jurkat cell line. The expression levels of TCR and HLA-I molecules were represented by the percentages of TCRα/β and HLA-A, B, C-positive cells respectively. (C) Expression of TCR and HLA-I on T cells following electroporation of Cas9 mRNA and sgRNAs twice. (D) TRAC-targeted and B2M-targeted gene disruptions were measured utilizing the mismatch-selective T7E1 surveyor nuclease assay on genomic DNA which was amplified from electroporated cells. Arrows indicated digested bands. M represented DNA markers. (E) Multiple peaks of Sanger sequencing results indicated CRISPR/Cas9-mediated NHEJ events at TRAC and B2M genes loci. (F) Indels and insertions were observed through single clonal sequence analysis of PCR amplification upon CRISPR/Cas9-mediated TRAC and B2M genes editing events.

**Figure S5.**

**
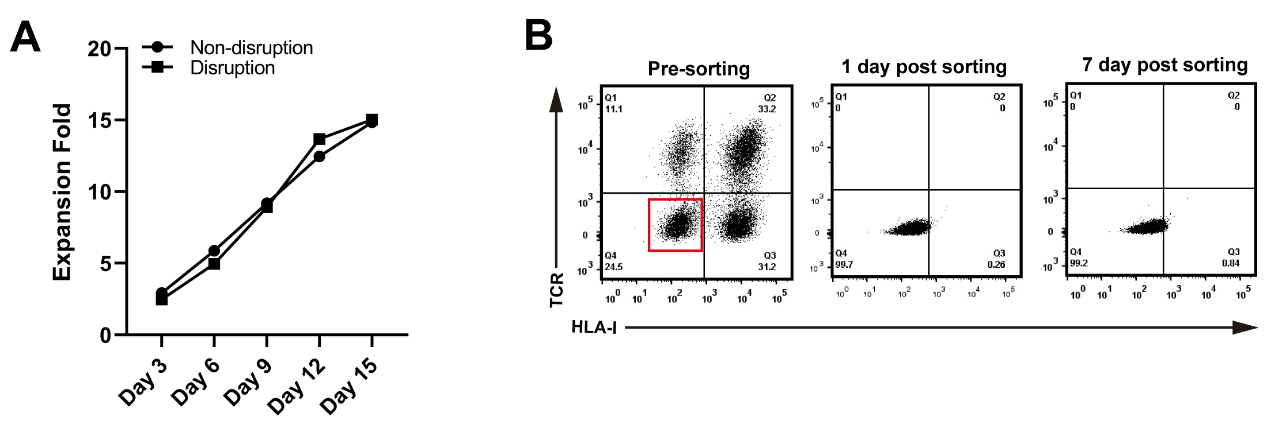
**

**Figure S5. Proliferation evaluation of electroporated T cells.**

(A)Relative expansion fold of electroporated T cells with or without targeted sgRNAs. (B) The expression of TCR and HLA-I was measured on electroporated T cells before sorting, one day post sorting and 7 days post sorting by flow cytometry.

**Figure S6**

**
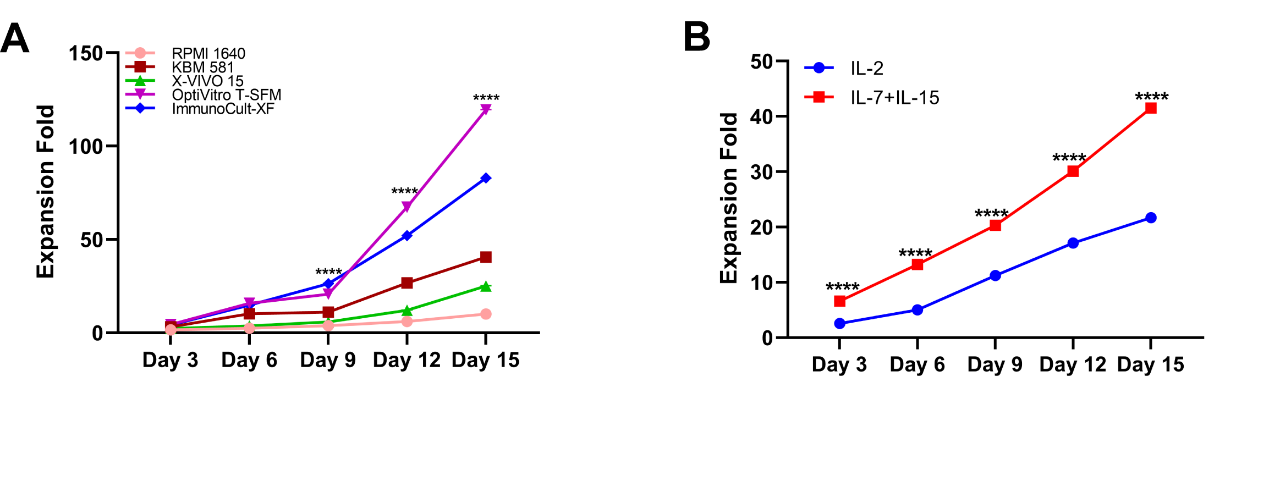
**

**Figure S6.** **Screening of culture media and cytokines.**

(A) Expansion of CD8^+^ T cells in various media including RPMI 1640, KBM 581, X-VIVO 15, OptiVitro T-SFM and ImmunoCult-X. (B) Expansion of α-CD3/α-CD28-activated CD8^+^ T cells in media containing cytokines of IL-2 or a combination of IL-7 and IL-15. Data represented as mean ± SD. Data in (A) were analyzed by two-way ANOVA with Tukey’s multiple comparisons test. Data in (B) were analyzed by two-way ANOVA with Sidak’s multiple comparisons test. **** *p* < 0.0001.

**Figure S7**

**
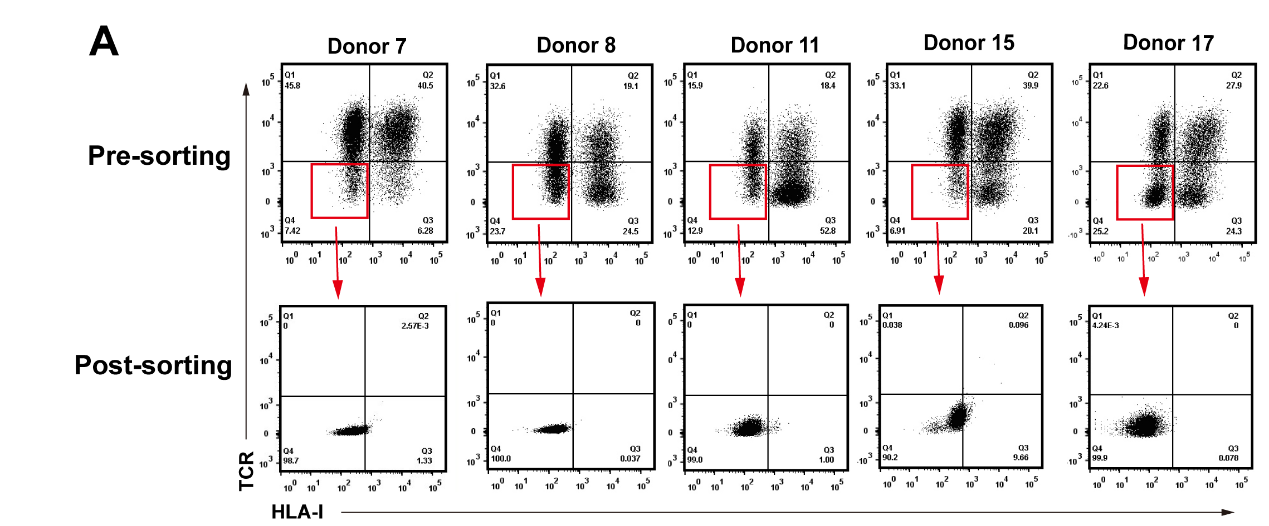
**

**Figure S7. Five dominant CD8^+^ T cells which were depleted of *TRAC* and *B2M*.**

(A) Five dominant CD8^+^ T cells were electroporated with *Cas9* mRNA and sgRNAs targeting *B2M* and *TRAC*. On Day 1, TCR and HLA-I double negative cells were sorted by flow cytometry. On Day 3, TCR^-^/HLA-I^-^ T cells were confirmed for the knockout efficiencies utilizing flow cytometry again.

**Figure S8**

**
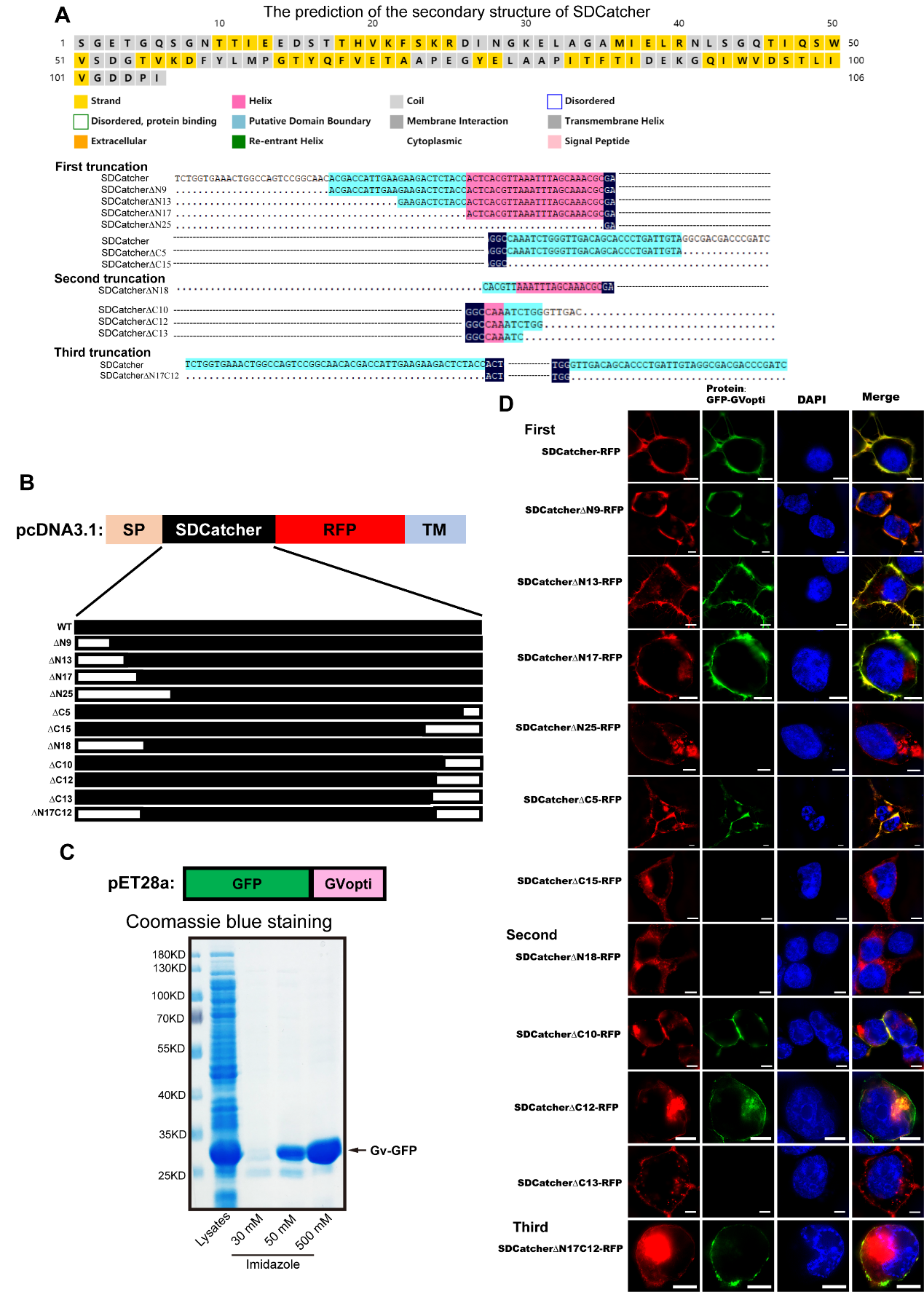
**

**Figure S8. The optimization of Sd without affecting conjugation capability to Gv.** (A) The strategy to truncate Sd to reduce its immunogenicity. (B) The schematic of truncated Sd variants. (C) Coomassie blue staining of purified Gv-GFP proteins. (D) HEK293T cells were transfected with various truncated Sd and subsequently incubated with Gv-GFP for 4 hours at 37 ℃. The immunofluorescence assay was used to evaluate their binding abilities. Red indicated the expression of truncated Sd on the cell membrane. Green represented the binding of Gv-GFP molecules after incubation. Scale bars represented 5 μm.

**Figure S9**


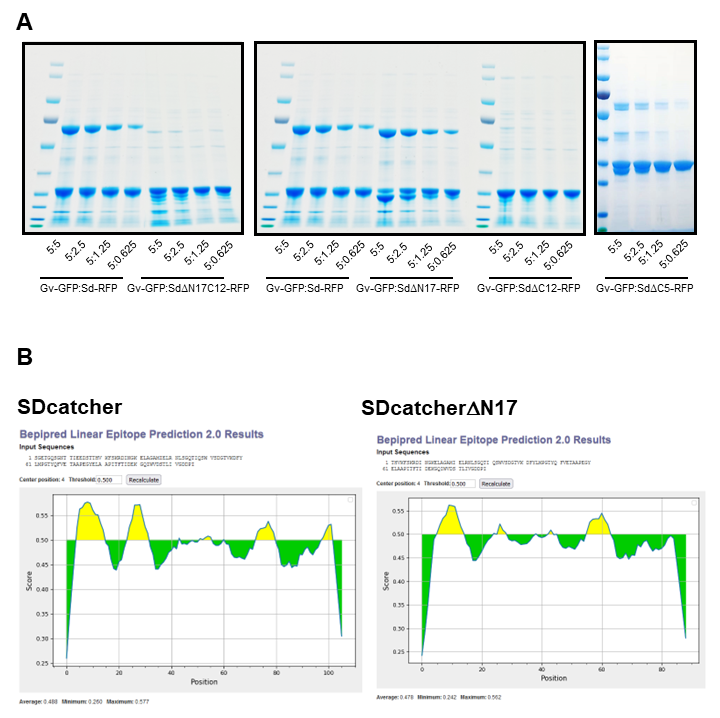


**Figure S9. Validation of the conjugation efficiency of optimized Sd variants.**

(A) The conjugation efficiency of Sd variants to Gv-GFP was evaluated by co-incubating various combinations of Sd variants with Gv-GFP, followed by the Coomassie blue staining assay. (B) Bepipred linear epitope prediction (Immune epitope database) of Sd and SdΔN17.

**Figure S10**

**
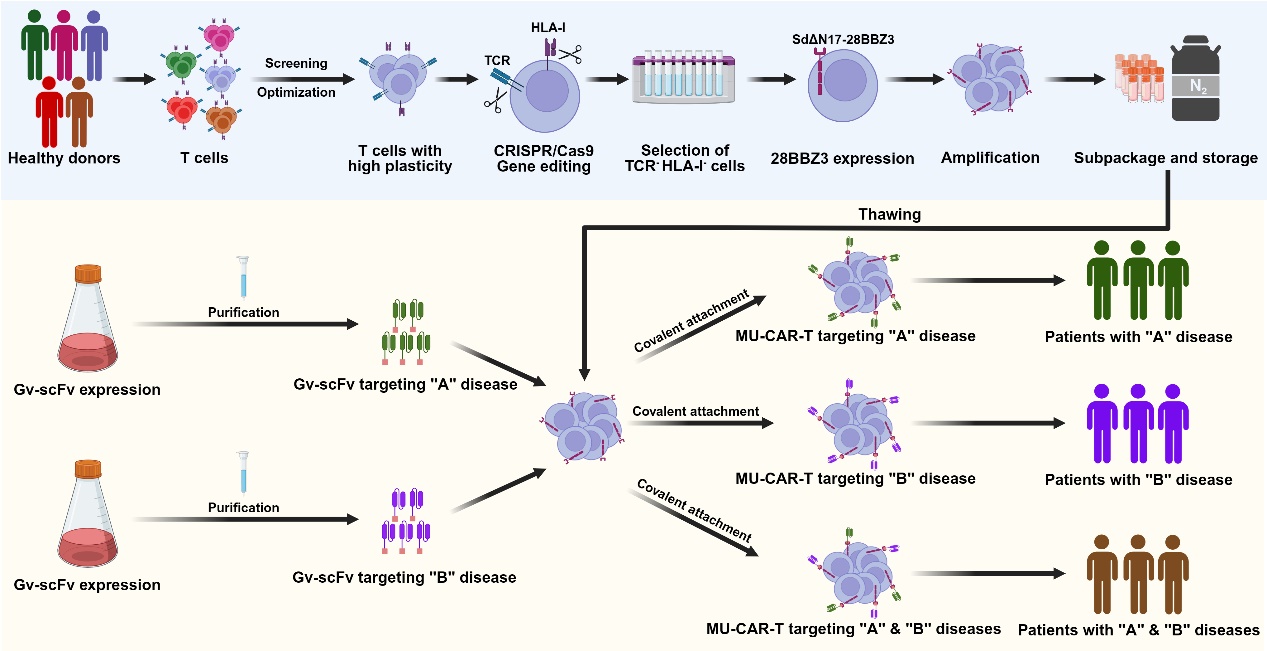
**

**Figure S10.** Manufacturing of MU-CAR-T cells. The manufacturing batch of MU-CAR-T cells had the potential to provide benefits for a diverse range of patients. The T cells with high plasticity were obtained by screening and optimization from different healthy donors. CRISPR/Cas9-mediated gene editing technique was subsequently utilized to eliminate TCR and HLA-I on T cells. SdΔN17-28BBZ3 was transduced into TCR^-^/HLA-I^-^ cells, followed by the enrichment, amplification, subpackage and storage of SdΔN17-28BBZ3-expressing population. MU-CAR-T cells were generated upon covalently linking to various Gv-tagged scFvs which were purified separately. The resulting diversified MU-CAR-T cells were administrated to patients with different diseases as a form of “live drug”.

Table S1. The sequences of related constructs.

| **Signal peptide (SP)** | MGILPSPGMPALLSLVSLLSVLLMGCVA |
| --- | --- |
| **28BBZ3** | GGGGSGGGGSGGGGSFPGPSKPFWVLVVVGGVLACYSLLVTVAFIIFWVRSKRSRLLHSDYMNMTPRRPGPTRKHYQPYAPPRDFAAYRSGGGGSKRGRKKLLYIFKQPFMRPVQTTQEEDGCSCRFPEEEEGGCELGGGGSKFSRSADAPAYQQGQNQLYNELNLGRREEYDVLDKRRGRDPEMGGKPQRRKNPQEGLYNELQKDKMAEAYSEIGMKGERRRGKGHDGLYQGLSTATKDTYDALHMQALPPR |
| **SdΔN17** | THVKFSKRDINGKELAGAMIELRNLSGQTIQSWVSDGTVKDFYLMPGTYQFVETAAPEGYELAAPITFTIDEKGQIWVDSTLIVGDDPI |
| **Gv** | KVGNTIVMVDKLKEVPTP |
| **VRC01 scFv** | MEIVLTQSPGTLSLSPGETAIISCRTSQYGSLAWYQQRPGQAPRLVIYSGSTRAAGIPDRFSGSRWGPDYNLTISNLESGDFGVYYCQQYEFFGQGTKVQVASDIKREGRGSLLTCGDVEENPGQVQLVQSGGQMKKPGESMRISCRASGYEFIDCTLNWIRLAPGKRPEWMGWLKPRGGAVNYARPLQGRVTMTRDVYSDTAFLELRSLTVDDTAVYFCTRGKNCDYNWDFEHWGRGTPVIVSS |
| **CD5-CD30 scFvs** | EIQLVQSGGGLVKPGGSVRISCAASGYTFTNYGMNWVRQAPGKGLEWMGWINTHTGEPTYADSFKGRFTFSLDDSKNTAYLQINSLRAEDTAVYFCTRRGYDWYFDVWGQGTTVTVSSGGGGSGGGGSGGGGSDIQMTQSPSSLSASVGDRVTITCRASQDINSYLSWFQQKPGKAPKTLIYRANRLESGVPSRFSGSGSGTDYTLTISSLQYEDFGIYYCQQYDESPWTFGGGTKLEIKGGGGSGGGGSGGGGSGGGGSQIQLQQSGPEVVKPGASVKISCKASGYTFTDYYITWVKQKPGQGLEWIGWIYPGSGNTKYNEKFKGKATLTVDTSSSTAFMQLSSLTSEDTAVYFCANYGNYWFAYWGQGTQVTVSAGGGGSGGGGSGGGGSDIVLTQSPASLAVSLGQRATISCKASQSVDFDGDSYMNWYQQKPGQPPKVLIYAASNLESGIPARFSGSGSGTDFTLNIHPVEEEDAATYYCQQSNEDPWTFGGGTKLEIK |
